# Influence of spatial characteristics of green spaces on microclimate in Suzhou Industrial Park of China

**DOI:** 10.1038/s41598-022-13108-1

**Published:** 2022-06-01

**Authors:** Xiangdong Xiao, Lulu Zhang, Yimei Xiong, Jiayi Jiang, Anqi Xu

**Affiliations:** 1 School of Architecture, Soochow University, Suzhou, 215123 China; 2 Xi’an Jiaotong-Liverpool University, Suzhou, 215123 China

**Keywords:** Climate-change ecology, Urban ecology

## Abstract

Continuous urban development leads to urban heat island effects. Research suggests that urban green spaces can help effectively reduce urban heat island effects in the summer. Previous studies have mainly focused on the influence of different underlying surfaces on air cooling and humidification. There is a lack of in-depth research on the relationship between park structure and microclimatic effects. Here, we examined the main landscape parameters of green spaces in 15 parks located in Suzhou Industrial Park (SIP) with a subtropical maritime monsoon climate zone during the summer to analyze their influence on the microclimate. We adopted a multiple regression method to perform a quantitative analysis of the correlation between the factors and the cooling and humidifying effects. We used one-way ANOVA (analysis of variance) and multiple linear regression statistical analysis methods to study the influence of woodland density and water bodies on the microclimatic effect of the green areas. The result shows that the average cooling and humidifying effect of medium-size green spaces was most significant during high-temperature hours in the daytime. Also, the result shows that the shape and size of water areas within a green space have a significant influence on local cooling and humidification.

## Introduction

Urban green spaces can produce cold island effects at various scales, thus effectively reducing urban heat island effects and improving the urban thermal environment. Existing studies suggest that the size of urban green spaces has a significant influence on the microclimate ^1–3^. Dimoudi et al. ^4^ found that the cooling and humidifying effect of parks increased with an increase in park size. However, Su et al.’s ^5^ and Chang et al.’s ^6^ studies on the relationship between park temperature and park size did not support previous research results. The studies of Chen et al. ^7^ suggested that the greater the park's size, the lower its internal temperature. The size of a park affects its temperature, and the location significantly affects the quantitative relationship between the temperature and size of a park ^7–10^. Which park size brings the best cooling and humidifying effect? Existing research on reducing the heat island effect by urban green spaces does not sufficiently focus on the correlation between park size and its cooling and humidifying effect. Kang et al. ^11^ analyzed the respective cooling and humidifying effects of four types of underlying surfaces: lawn, woodland, bare land, and cement. They showed that both woodland and lawn produced a cooling and humidifying effect in high temperatures in summer, with woodland having the greatest cooling and humidifying effect ^11,12^. Burkart et al. ^13^ used remote sensing data and geographic information to determine the distance between urban vegetation and water bodies, taking into account fit Poisson generalized additive models and the interaction temperature among equivalent variables. Their result suggested that urban green spaces and blue spaces reduce the heat-related mortality of urban vegetation. Summarily, green spaces of different sizes vary significantly in their cooling and humidifying effect. Furthermore, woodland and water areas within lakes and nearby green spaces also have a significant cooling and humidifying effect. However, previous studies mainly focused on the influence of different underlying surfaces on the cooling and humidifying effect. There is a lack of in-depth research of the cooling and humidifying effect of green spaces of different sizes. Also, the influence of the density of woodland and water areas within green spaces on the cooling and humidifying effect with regard to underlying surfaces is understudied. In this study, the researched sample was Suzhou Industrial Park (SIP), located ≈200 km away from the ocean. Therefore, the influence of the ocean was minor and could be omitted. The lakes near the researched sample were not large and > 20 km away. Therefore, the cooling and humidifying effect of the lakes were minor and could also be omitted. We aimed to study the influence of woodland density and structure of water areas in green spaces on their local cooling and humidifying effects. We measured the trends of average daily changes of cooling and humidifying effects of green spaces of different sizes, as well as the relationship between various structure parameters of green spaces (i.e., perimeter, area, perimeter-area ratio, leaf area index, and canopy density) and the cooling and humidifying effects. Such parameters are key factors that influence the internal and external thermal environment of parks ^2,14–17^. This study can guide the planning of parks by optimizing the layout of woodlands and water bodies, to enhance the cooling and humidifying effects of green spaces in hot summer conditions, improve the urban microclimate and contribute to the sustainable development of cities.

In this study, we first analyzed the cooling and humidifying effects of green spaces of different sizes in summer. We adopted a multiple regression method to perform a quantitative analysis of the correlation between the factors (i.e., green space area, perimeter, perimeter-area ratio, leaf area index, and canopy density) and the cooling and humidifying effects. We then further divided the green spaces according to their structure: green spaces a short, medium, and long distance from water and green spaces with high and low-density woodland, to analyze the correlation between the structure of the spaces and their microclimatic effects. We used one-way ANOVA (analysis of variance) and multiple linear regression statistical analysis methods to study the influence of woodland density and water bodies on the microclimatic effect of the green areas. Finally, we studied the influence of three typical shapes of water areas of increasing size on the cooling and humidifying effects through numerical simulations. The research results will further improve and supplement the urban green space heat island effect research and evaluation system and provide practical guidance for the construction of urban ecological infrastructure.

## Materials and methods

### Study area sample choice and measurement point setting

The urban planning of SIP itself is different from that of other regions in China. In its planning and organizational layout, a clear hierarchical layout is adopted, referring to the planning principles of Singapore’s neighborhood center. It proposes an organizational concept for the planning of public facilities at the regional, zoning, and neighborhood levels. On this basis, SIP divides the park green space into three levels, namely city-level parks, district-level parks, and neighborhood parks. In these three levels, different land use is set up according to the size of the service population. This research mainly studies a total of 29 city-level and district-level parks in SIP, which are representative, and the total sample size is limited.

**Figure 1:**
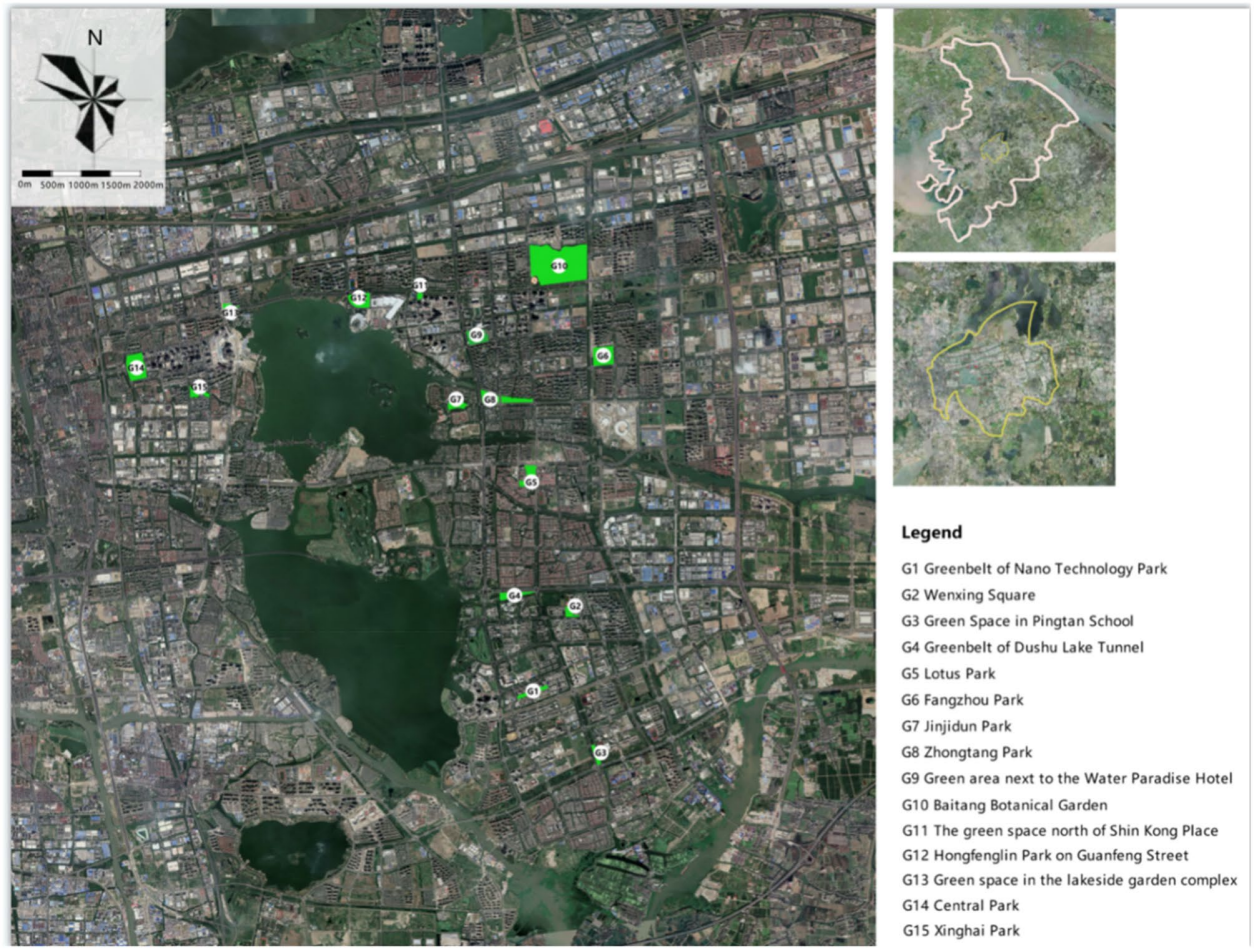
Plot layout and geometry (Source: Maps Data: Baidu©2022 DigitalGlobe, Technologies).

**Figure 2:**
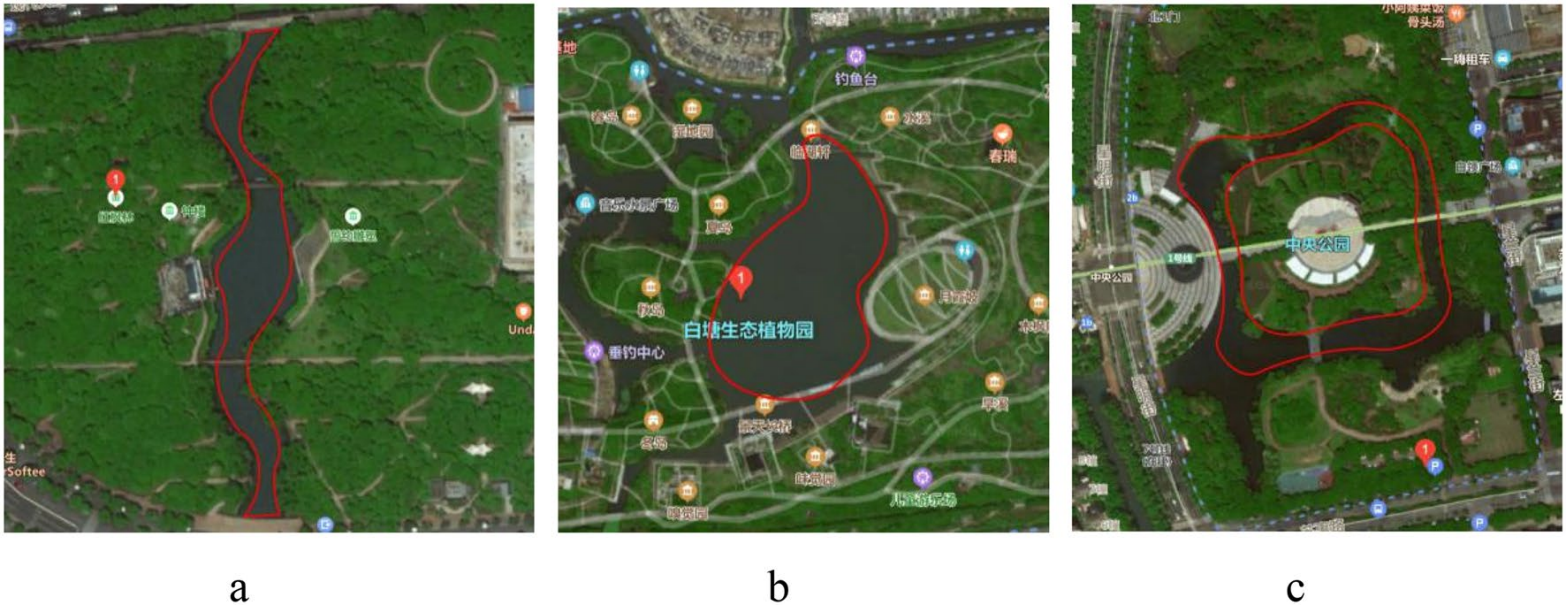
Satellite images and numerical simulations of representative water shapes. (**a**) Banded water (Hongfenglin Park); (**b**) massive water (Baitang Botanical Garden); (**c**) annular water (Central Park) ( https://map.baidu.com/search/%E8%8B%8F%E5%B7%9E%E4%B8%AD%E5%A4%AE%E5%85%AC%E5%9B%AD/@13432986.830403794,3651910.902291707,17.87z/maptype%3DB_EARTH_MAP?querytype=s&da_src=shareurl&wd=%E8%8B%8F%E5%B7%9E%E4%B8%AD%E5%A4%AE%E5%85%AC%E5%9B%AD&c=1&src=0&pn=0&sug=0&l=7&b=(12751614.672841096,3237606.544511262;15633781.7749508,4644164.135488758)&from=webmap&biz_forward=%7B%22scaler%22:1,%22styles%22:%22pl%22%7D&device_ratio=1).

Based on UAV (Unmanned Aerial Vehicle; DJI MAVIC) aerial photos with a perspective elevation of 150 m, and previous research on parks and green spaces in SIP, we selected 15 green spaces in parks as the research objects. The selection of our research objects was mainly based on the following aspects. First, considering the size range of the 15 green park spaces, and by reference to previous research reports, we adopted 4 ha and 10 ha as the critical point in defining small, medium, and large size green spaces ^18,19^ to guarantee that the spaces were comparable. The 15 green spaces were divided into small-size green spaces (area < 4 ha), medium-size green spaces (area = 4–10 ha) and large-size green spaces (area > 10 ha). Secondly, the total number of parks and green spaces in SIP is 29. We divide the 29 parks and green spaces into three types: large, medium, and small. Using the method of stratified sampling, the research subjects were independently and randomly selected from 29 parks, and finally 15 park green spaces were obtained. Stratified sampling, also known as stratified extraction method, is a statistical method of sampling samples from a statistical population (also known as "population") ^20^. The sampling unit is divided into different layers according to a certain characteristic or a certain rule, and then samples are drawn independently and randomly from different layers. This ensures that the structure of the sample is relatively like the overall structure, thereby improving the accuracy of the estimation. The spatial characteristics of the 15 parks after sampling are summarized as follows: the green coverage rate ranged from 28.73% to 94.35%, the canopy density ranged from 0.21 to 0.85, and the selected green spaces had water bodies of different sizes. Within each green space, we distinguished between green spaces a short, medium, or long distance from water, sparse forest (canopy density range of 0.4 to 0.6), dense forest (canopy density range of 0.7 to 1.0) and other patches. Green spaces a short/medium/long distance from water were classified based on the distance between the boundary of the green space and that of the water body inside the green space. Since the distance between the green space boundary and that of the water bodies differed, green spaces a short/medium/long distance from water were determined based on the distance ratio. For example, if the distance between the boundary of the water body and that of the green space within the researched sample is 200 m (i.e., the ratio s 1), then a green space which is 50 m (ratio of 1/4) away from the boundary of the water body is defined as a green space a short distance from water. A green space which is 100 m (ratio of 1/2) away from the water body boundary is defined as a green space a medium distance from water. A green space which is 150 m (ratio of 3/4) away from the boundary of the water body is defined as a green space a long distance from water. Each research sample was defined as a circular area with a radius of 10 m. In each research sample, three observation points were distributed evenly. Figure 1 and Table 1 show the detailed characteristics of the 15 green spaces. In the numerical simulation study on the effect of the shape and area of the water body, the shapes of water bodies in the 15 green spaces were classified into representative and typical banded water, massive water, and annular water (see Fig. 2).

**Table 1:**
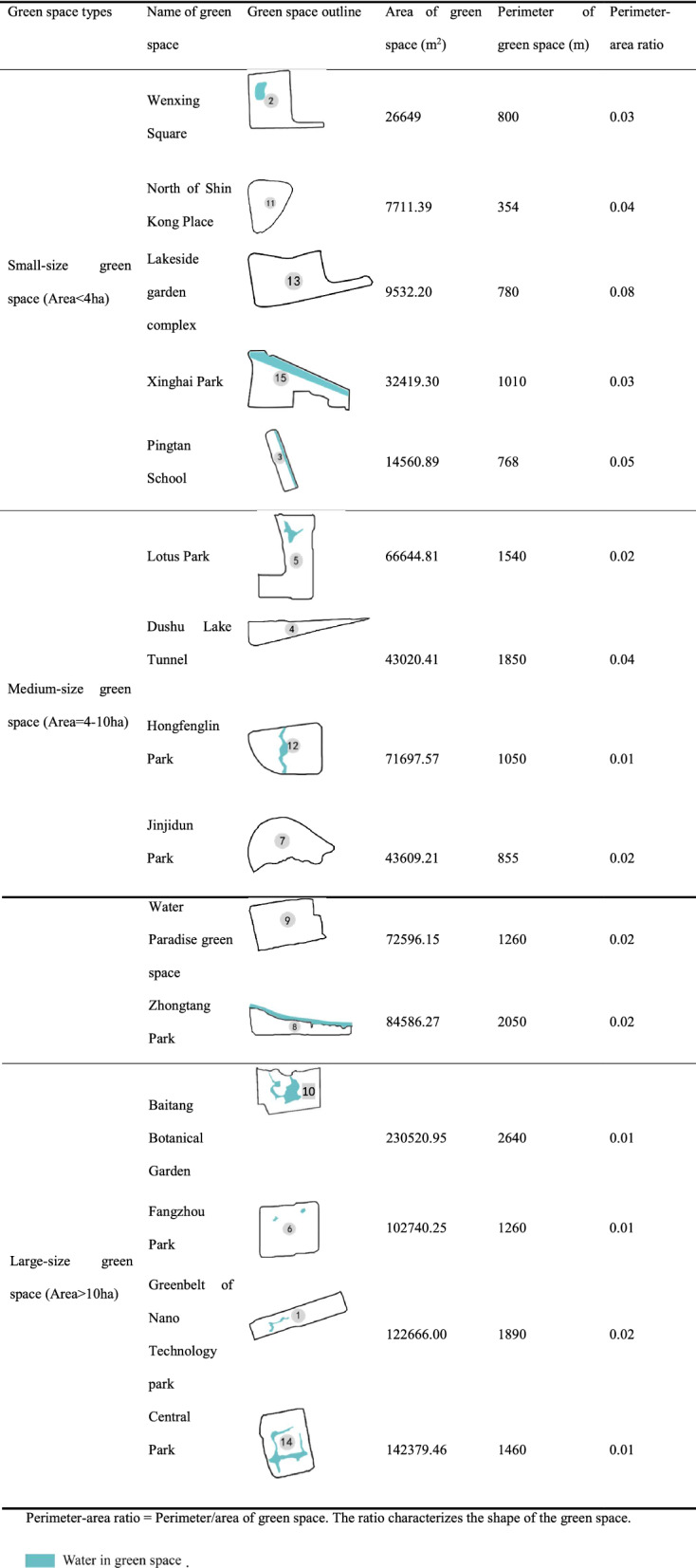
Green space outline and structural geometry.

### Measurements of variables

This experiment was conducted under sunny and windless (wind speed < 2 m/s) weather conditions from July to August 2018 and July to August 2019. Observations were taken during the 10:00–16:00 period. This study used a RC-4HC Temperature and Humidity automatic recorder (accuracy: ± 0.5 °C TEMP; ± 3%RH) to measure air temperature and humidity. The recorder was placed under the forest canopy 1.5 m from the ground and recorded the temperature and humidity data every 10 s. We used a CI-110 Plant Canopy Imager (CID Bio Science, Inc.) to measure the leaf area index of plants and other indicators. We used UAV (DJI MAVIC) aerial photos and AutoCAD2016 (Autodesk Computer Aided Design, Autodesk, Inc.) to calculate the perimeter and area of each green space.

### Statistical analyses

Previous research suggested that urban cold islands, formed by parks consisting mainly of green spaces and water bodies, effectively mitigated the urban heat island effect and improved the urban thermal environment. With a greater area of green space and water bodies, it is easier to form strong local circulation, producing a greater effect on the surrounding thermal effect. Therefore, there is a significant positive correlation between the cooling effect, the area of woodland, and water bodies, which are the key factors that influence the internal and external thermal environment of a park. In this research, we first calculated the average value of temperatures and humidity at each hour between 10:00 and 14:00 at the three observation points. This procedure was carried out for all small-size, medium-size, and large-size green spaces. Then, by processing the temperature and humidity data, we obtained the cooling and humidifying intensity at each site. The three repeated contrast points were located at an empty area about 100 m away from the sampling points. We used the SPSS22.0 software to analyze correlations between the perimeter, area, perimeter-area ratio, leaf area index, canopy density, and other structural characteristics of the green spaces and their cooling and humidifying effects to construct a prediction model. The formula for leaf area index (LAI) is given as Eq. (1)

1 $$LAI = In\tau_{\varphi i} / - K_{\varphi i}$$

In the formula, LAI represents the leaf area index, $\tau_{\varphi }$ represents the direct radiation transmission (or visible sky ratio) coefficient in each zenith angle area, $\tau_{\varphi i}$ represents the ith zenith angle division, and K represents the extinction coefficient of the canopy. The calculation formula for canopy density is expressed as Eq. (2):

2 $$CD = 1 - \beta /\alpha$$

where CD represents the canopy density, β represents the sky pixel value of the plant canopy, and α represents the pixel value of the plant canopy. We then used Duncan's ^21^ method of multiple comparisons to examine the differences in the cooling and humidifying intensity of the green spaces (a short/medium/long distance from water), thus analyzing the influence of water bodies on the cooling and humidifying effect of green spaces. We also adopted numerical modeling to research further the cooling and humidifying effect of banded water, massive water, and annular water. We defined the cooling intensity (cooling effect) as the difference between the air temperature at the contrast point and the average air temperature at each research point. The calculation is shown in Eq. (3):

3 $$\Delta T = t_{1} - t_{2}$$

where $t_{1}$ represents the air temperature at the contrast point, and $t_{2}$ represents the average air temperature at each measurement point in the sample site. ∆T, the difference between the above temperatures, is defined as the cooling effect. We defined the humidifying intensity (humidifying effect) as the difference between the relative humidity of the contrast point and the average air relative humidity of each measurement point. The calculation is shown in Eq. (4):

4 $$\Delta H = h_{1} - h_{2}$$

where $h_{1}$ represents the average air humidity of each measurement point in the green space, and $h_{2}$ represents the air humidity of the contrast point. ∆H, the difference between humidities, is defined as the humidifying effect ^22^.

**Figure 3:**
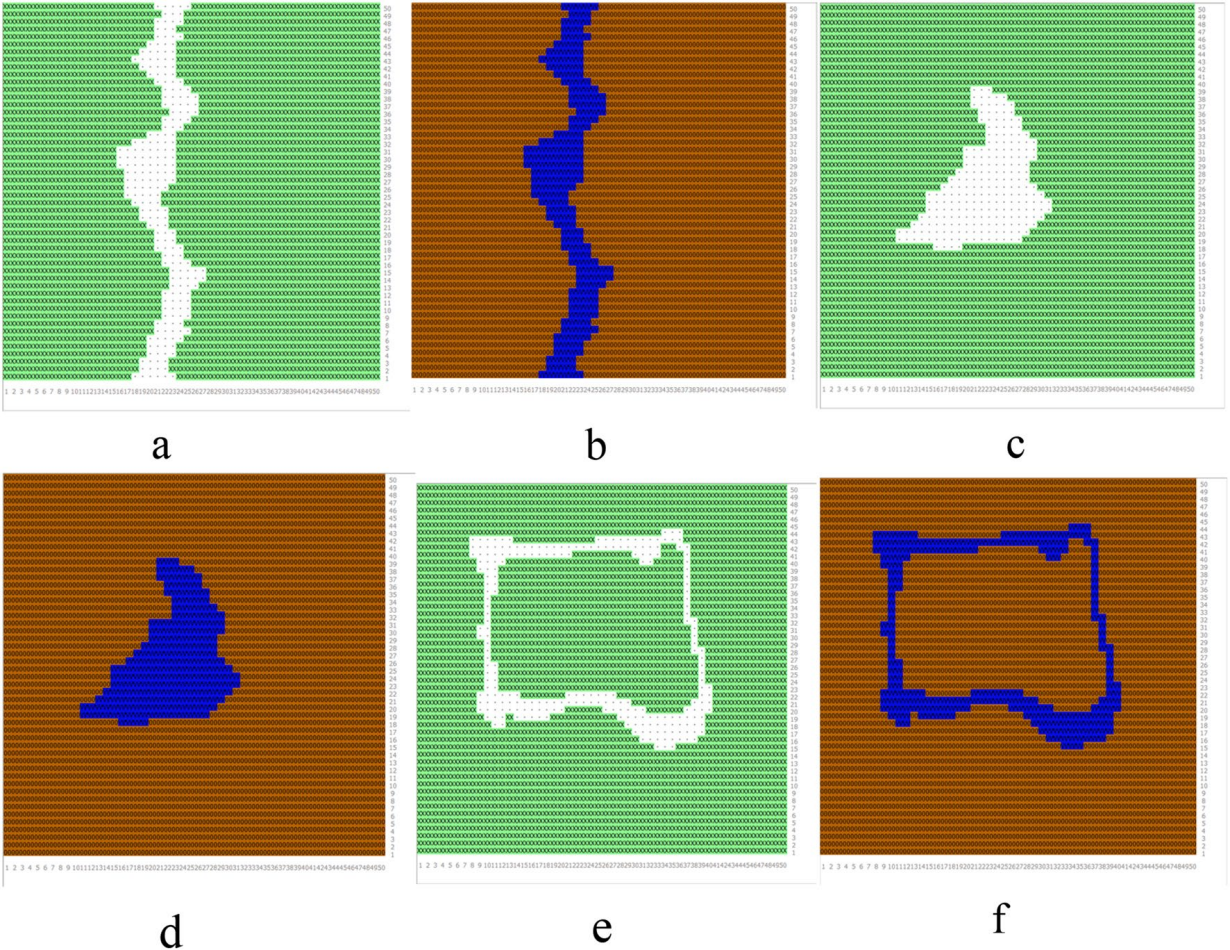
ENVI-met model at original site and with 5% and 10% increase of water area. (**a**) Banded water vegetation model; (**b**) Banded water soil surface model; (**c**) Massive water vegetation model; (**d**) Massive water soil surface model; (**e**) Annular water vegetation model; (**f**) Annular water soil surface model.

We used a numerical simulation method to study the effects of increasing the area of water bodies by 5% and 10%, and the effect of water shape (banded water, massive water, and annular water areas) on cooling and humidification. Figure 3 shows the simulation images in a 150 × 150 m area. Considering the characteristics of the simulation software and of the measurement site, the model had a total of 50 × 50 × 40 grids, and the grid resolution was 3 × 3 m. The parameter settings of this simulation input are presented in Table 2. In this study, the Root Mean Square Error (RMSE) and Mean Absolute Percentage Error (MAPE) of air temperature and relative humidity were 0.14 °C and 0.95% and 1.56% and 2.69%, respectively.

**Table 2:** Input parameter setting in simulations.

Parameter	Setting
Start time of simulation	7:00 on 18 July, 2019
Simulation time	12 h
Simulated initial temperature/relative humidity	31.13 °C/78.75% (The measured value of the day)
Wind direction/speed	northeastern/5.5 m/s
Roughness length	0.01

The multiple regression and numerical simulation models of the cooling and humidifying effects presented above produced statistically significant results. They constitute a useful method to evaluate and predict the cooling and humidifying effects of different types of green spaces. For data processing and statistical analysis in this experiment, we used SPSS22.0 (SPSS Inc.) software. For the numerical simulation, we used the ENVI-met software, while we used Microsoft Excel2019 and Origin8.0 software to produce the charts.

## Results

In this study, the five main characteristics of green spaces that were measured were area, perimeter, perimeter-area ratio, leaf area index, and canopy density. The structure of parameter between them is shown in Table 3.

**Table 3:** Parameter structure of the cooling and humidification effect based on the spatial characteristics of green spaces.

Content	Indicator name	Specific parameters
Correlation between the spatial characteristics of green spaces and the intensity of cooling and humidification	Meteorological factors	Cooling intensity
		Humidifying intensity
	Spatial characteristics of green space	Area
		Perimeter
		Perimeter-area ratio
		Leaf area index
		Canopy density

### Correlation between various spatial characteristics and cooling and humidifying intensity in green spaces

#### Small-size green spaces

**Figure 4:**
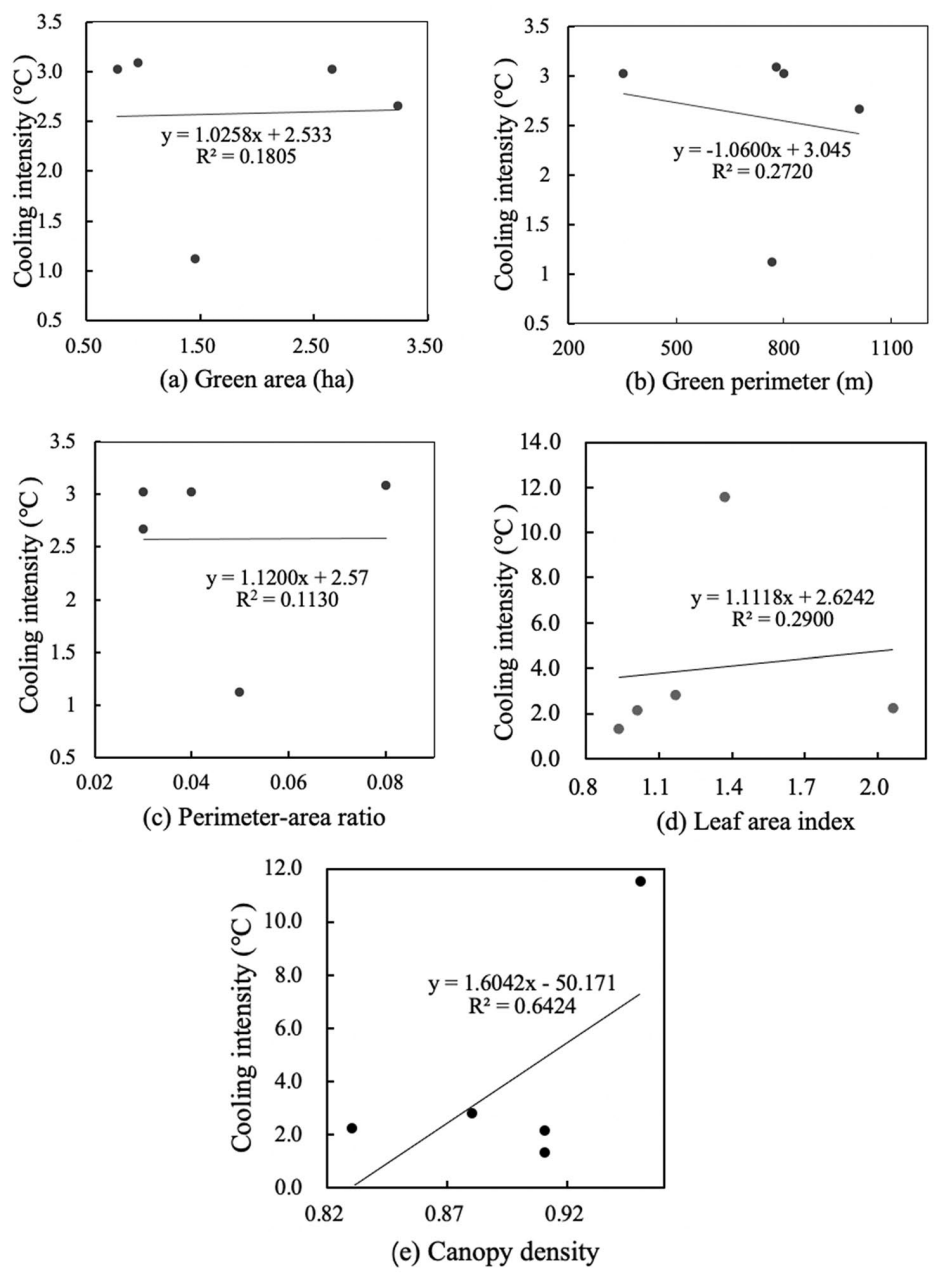
Linear regressions between spatial characteristics and cooling intensity of small-size green spaces.

**Figure 5:**
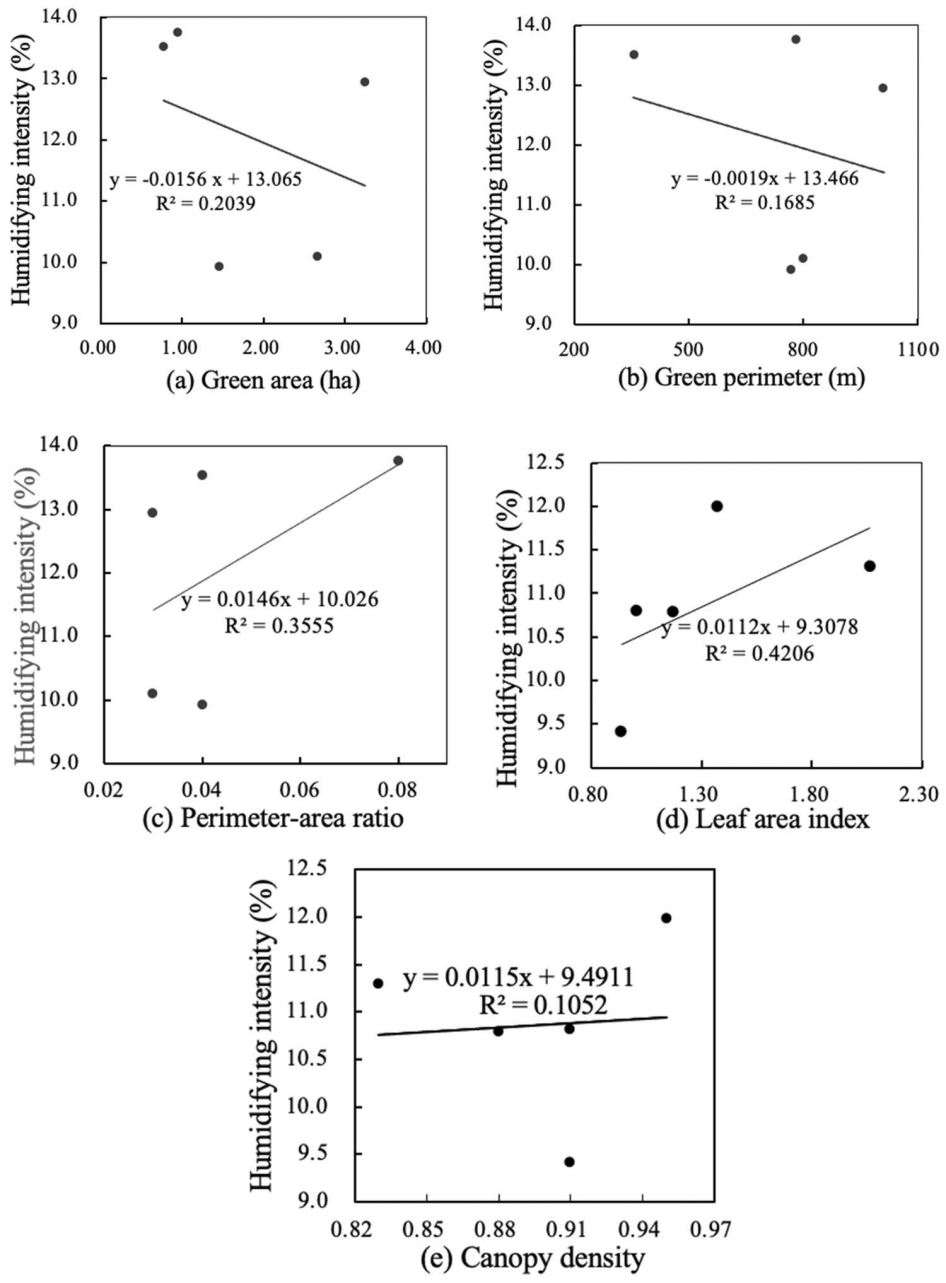
Linear regressions of spatial characteristics and humidifying intensity of small-size green spaces.

**Figure 6:**
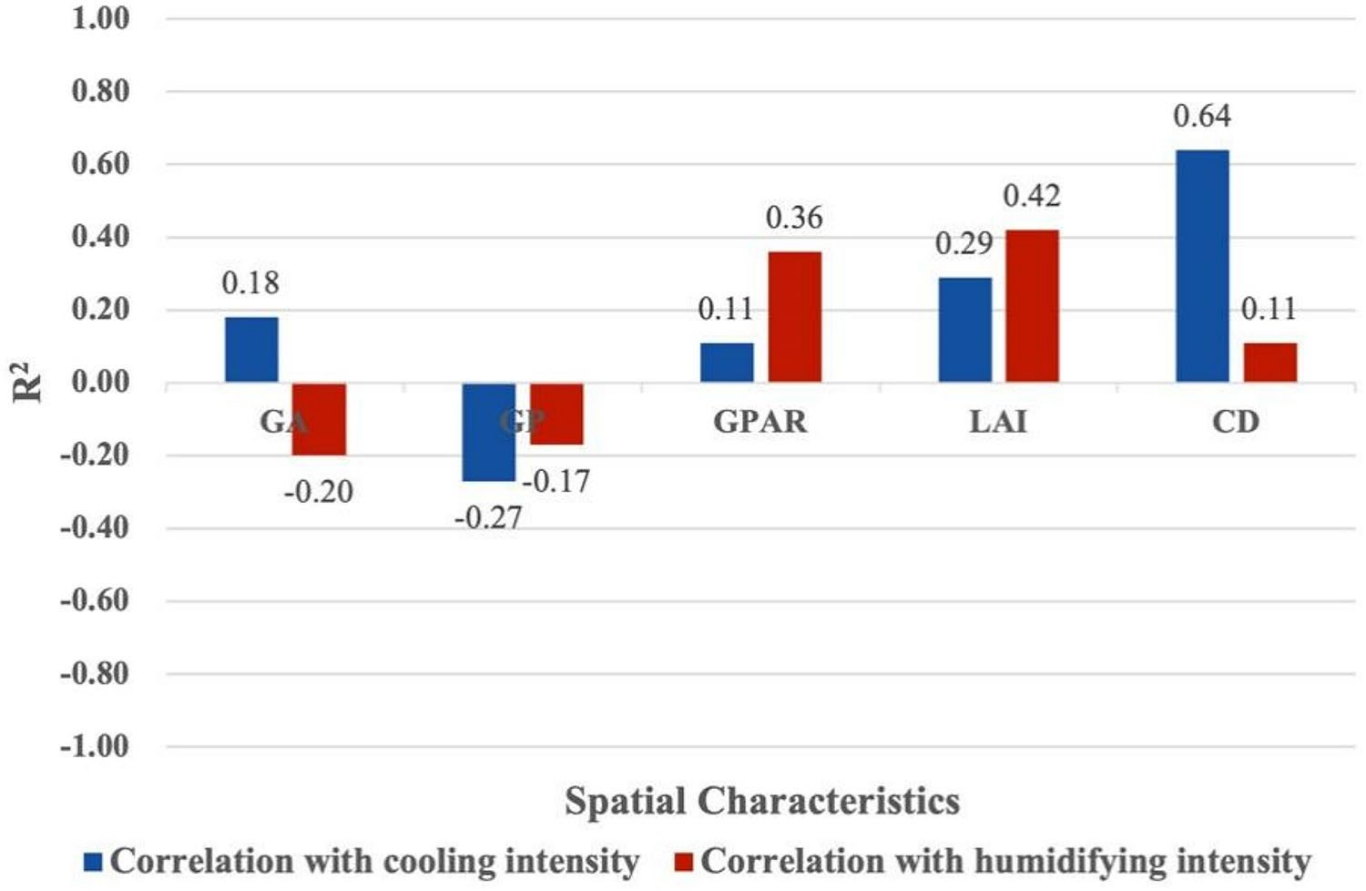
The correlation between the spatial characteristics of small-size green spaces and the intensity of cooling and humidifying (GA means green area; GP means green perimeter; GPAR means green perimeter-area ratio; LAI means leaf area index; CD means canopy density).

Figures 4 and 6 shows the results of linear regressions between spatial characteristics and the cooling effect in small-size green spaces. There were relatively weak correlations between area, perimeter, perimeter-area ratio, leaf area index and cooling intensity, and a strong correlation between canopy density and cooling intensity. Small-size green space has the weakest positive correlation between perimeter-area ratio and cooling intensity (R^2^ = 0.11), and its canopy density and cooling intensity have the strongest positive correlation (R^2^ = 0.64). Meanwhile, small-size green space has weakest negative correlation between perimeter and humidifying intensity (R^2^ = 0.17), and its leaf area index and humidifying intensity have significant positive correlation (R^2^ = 0.42). Figures 4a and 5a show that for every 1 ha increase in area of small-size green spaces, the cooling intensity increased by 1.026 °C, and the humidifying intensity decreased by 1.56%. Figures 4b and 5b show that for every 100 m increase in perimeter, the cooling intensity decreases by 1.06 °C, and the humidifying intensity decreased by 1.19%. Figures 4c and 5c show that for every 0.01 increase in the perimeter-area ratio, the cooling intensity increases by 1.12 °C, and the humidifying intensity increased by 1.46%. Figures 4d and 5d show that for every 0.1 increase in the leaf area index, the cooling intensity increases by 1.11 °C, and the humidifying intensity increased by 1.12%. Figures 4e and 5e show that each 0.01 increase in the canopy density, the cooling intensity increases by 1.60 °C, and each 0.1 increase in canopy density, the humidifying intensity increased by 1.15% (Fig. 6).

#### Medium-size green spaces

**Figure 7:**
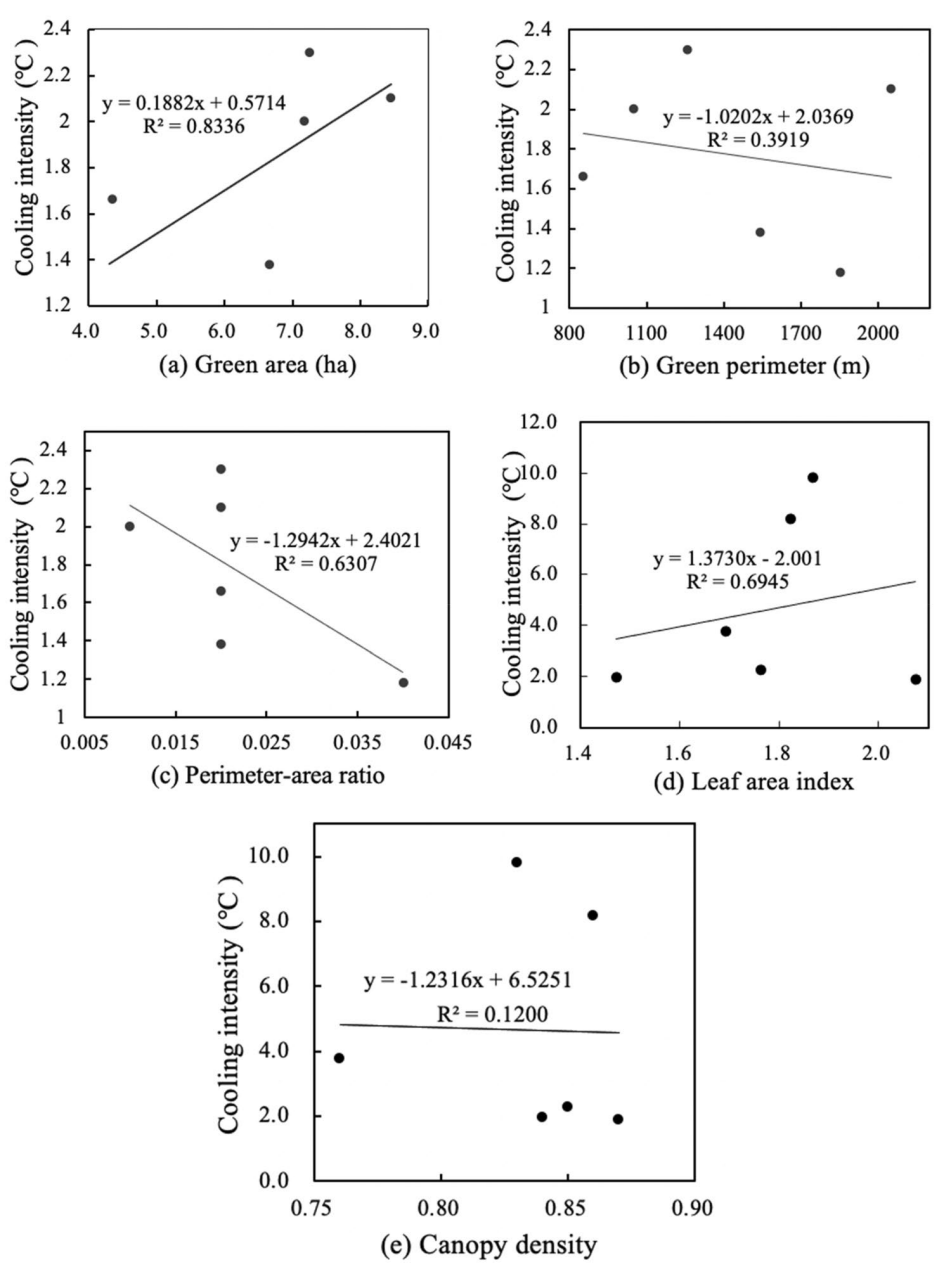
Linear regressions between spatial characteristics and cooling intensity of medium-size green spaces.

**Figure 8:**
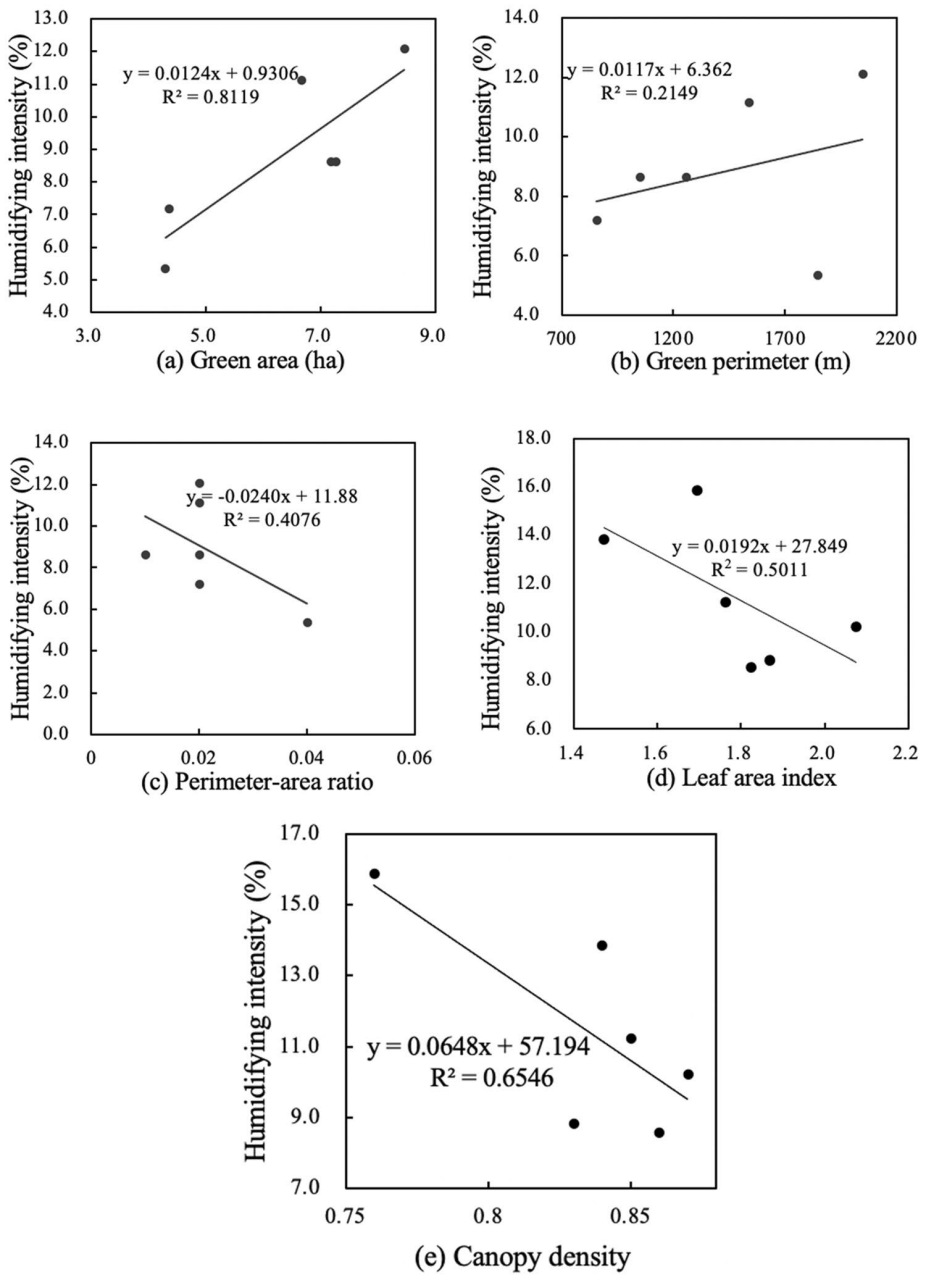
Linear regressions of spatial characteristics and humidifying intensity of medium-size green spaces.

**Figure 9:**
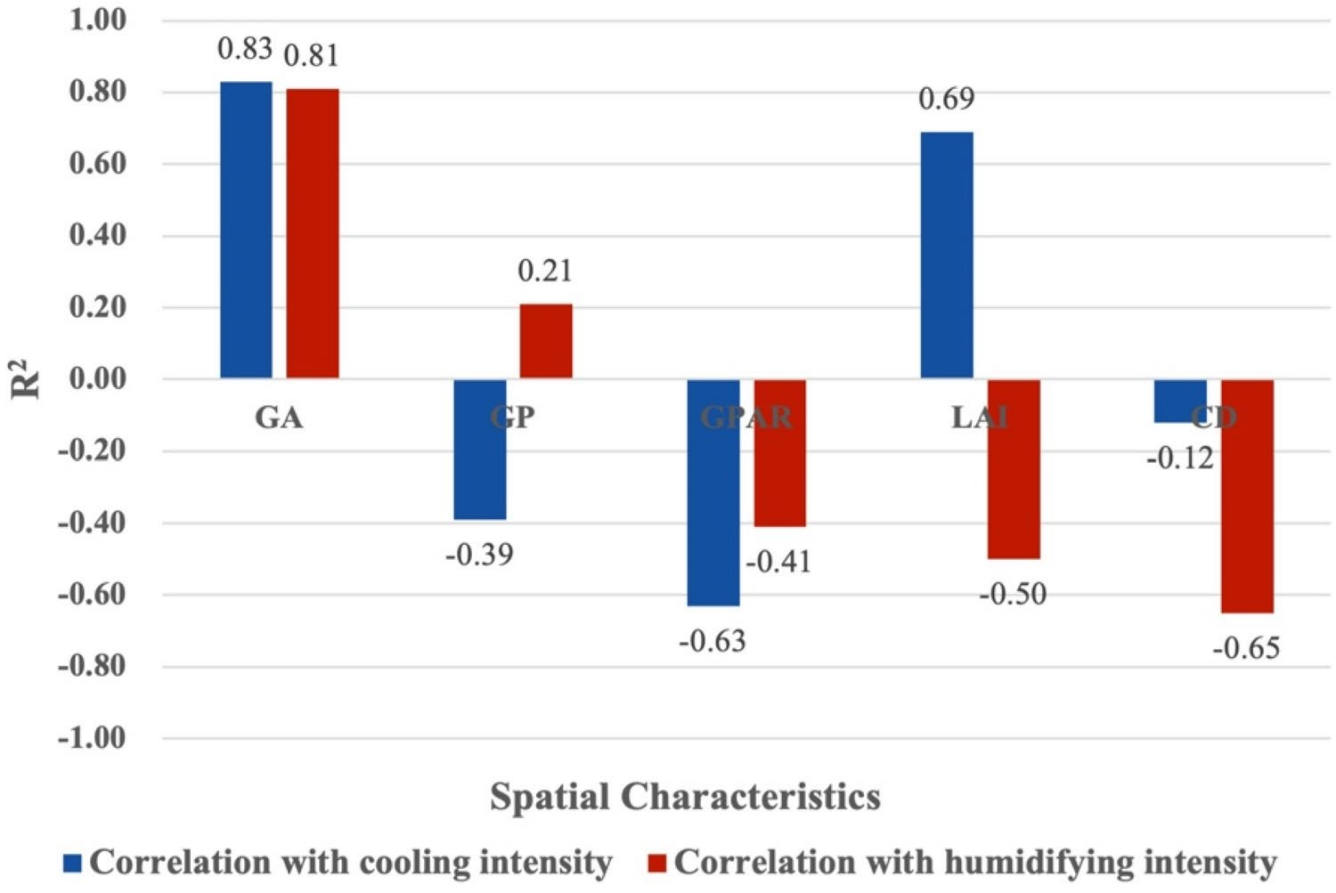
The correlation between the spatial characteristics of medium-size green spaces and the intensity of cooling and humidifying (GA means green area; GP means green perimeter; GPAR means green perimeter-area ratio; LAI means leaf area index; CD means canopy density).

Figures 7 and 9 shows the linear regressions between spatial characteristics and cooling intensity in medium-size green spaces. There was an extremely significant positive correlation between area and cooling intensity, an insignificant positive correlation between the leaf area index and cooling intensity, and a relatively weak negative correlation between the other three characteristics and cooling intensity. Medium-size green space has the weakest negative correlation between canopy density and cooling intensity (R^2^ = 0.12), and its green area and cooling intensity have the strongest positive correlation (R^2^ = 0.83). Meanwhile, medium-size green space has weakest negative correlation between perimeter-area ratio and humidifying intensity (R^2^ = 0.41), and its area and humidifying intensity have most significant positive correlation (R^2^ = 0.81). Figures 7a and 8a show that for every 1 ha increase in area of medium-size green spaces, the cooling intensity increased by 1.19 °C, and the humidifying intensity increased by 1.24%. Figures 7b and 8b show that for every 100 m increase in perimeter, the cooling intensity decreases by 1.02 °C, and the humidifying intensity increased by 1.17%. Figures 7c and 8c show that for every 0.01 increase in the perimeter-area ratio, the cooling intensity decreases by 1.29 °C, and the humidifying intensity decreased by 2.40%. Figures 7d and 8d show that for every 0.1 increase in the leaf area index, the cooling intensity increases by 1.37 °C, and the humidifying intensity decreased by 1.92%. Figures 7e and 8e show that each 0.01 increase in the canopy density, increases the cooling intensity decreases by 1.23 °C, and the humidifying intensity decreased by 6.48% (Fig. 9).

#### Large-size green spaces

**Figure 10:**
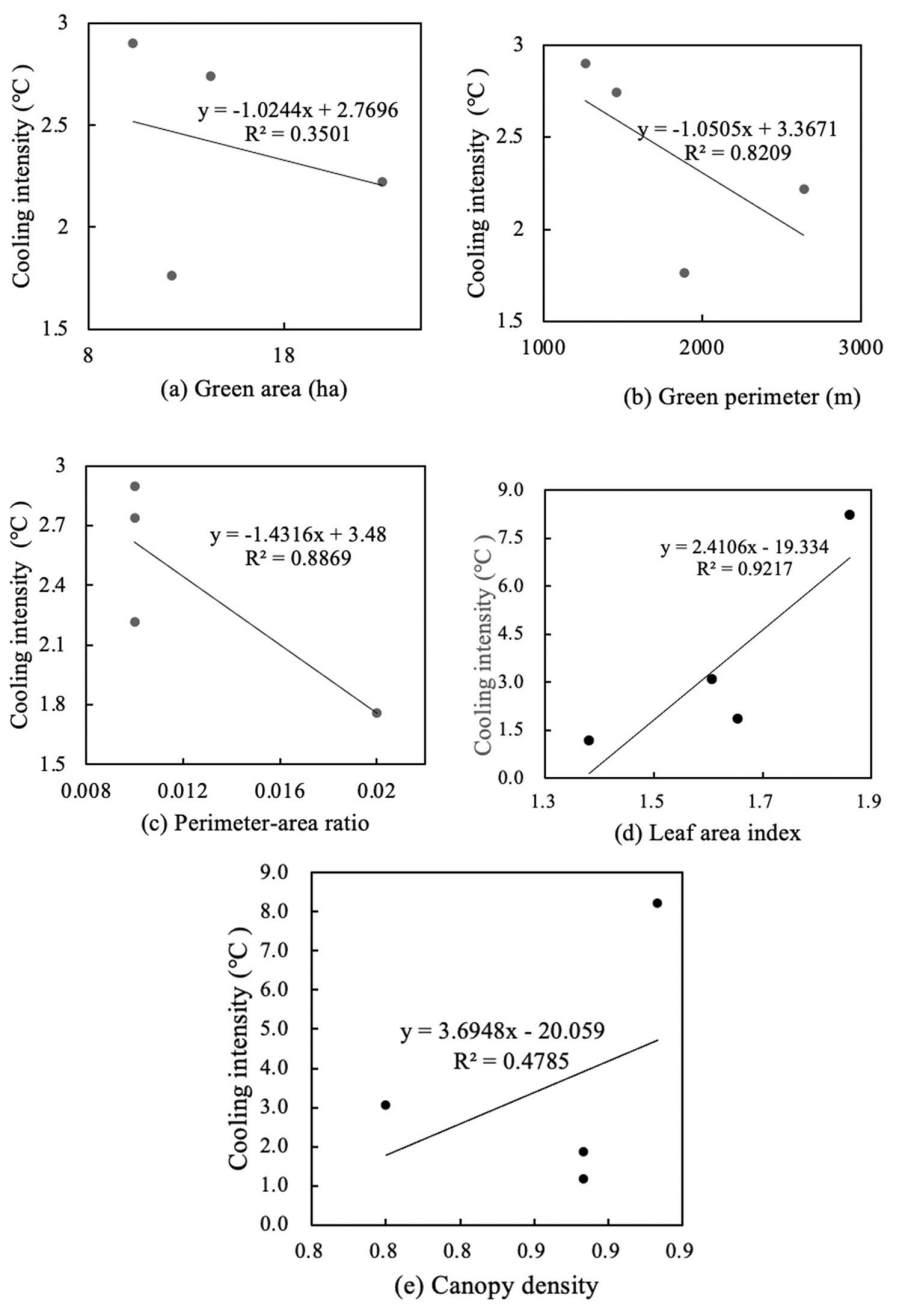
Linear regressions of spatial characteristics and cooling intensity of large-size green spaces.

**Figure 11:**
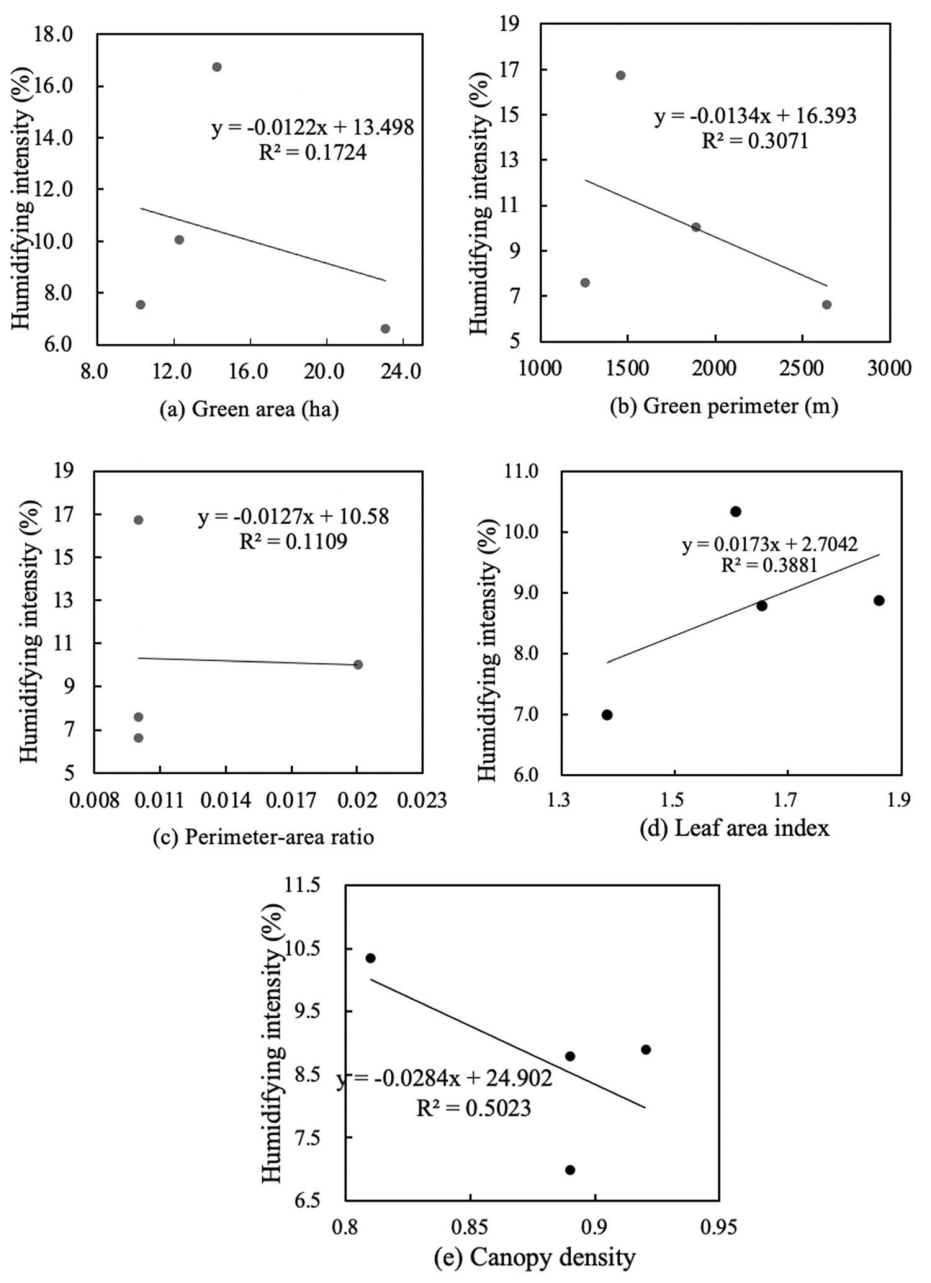
Linear regressions of spatial characteristics and humidifying intensity of large-size green spaces.

**Figure 12:**
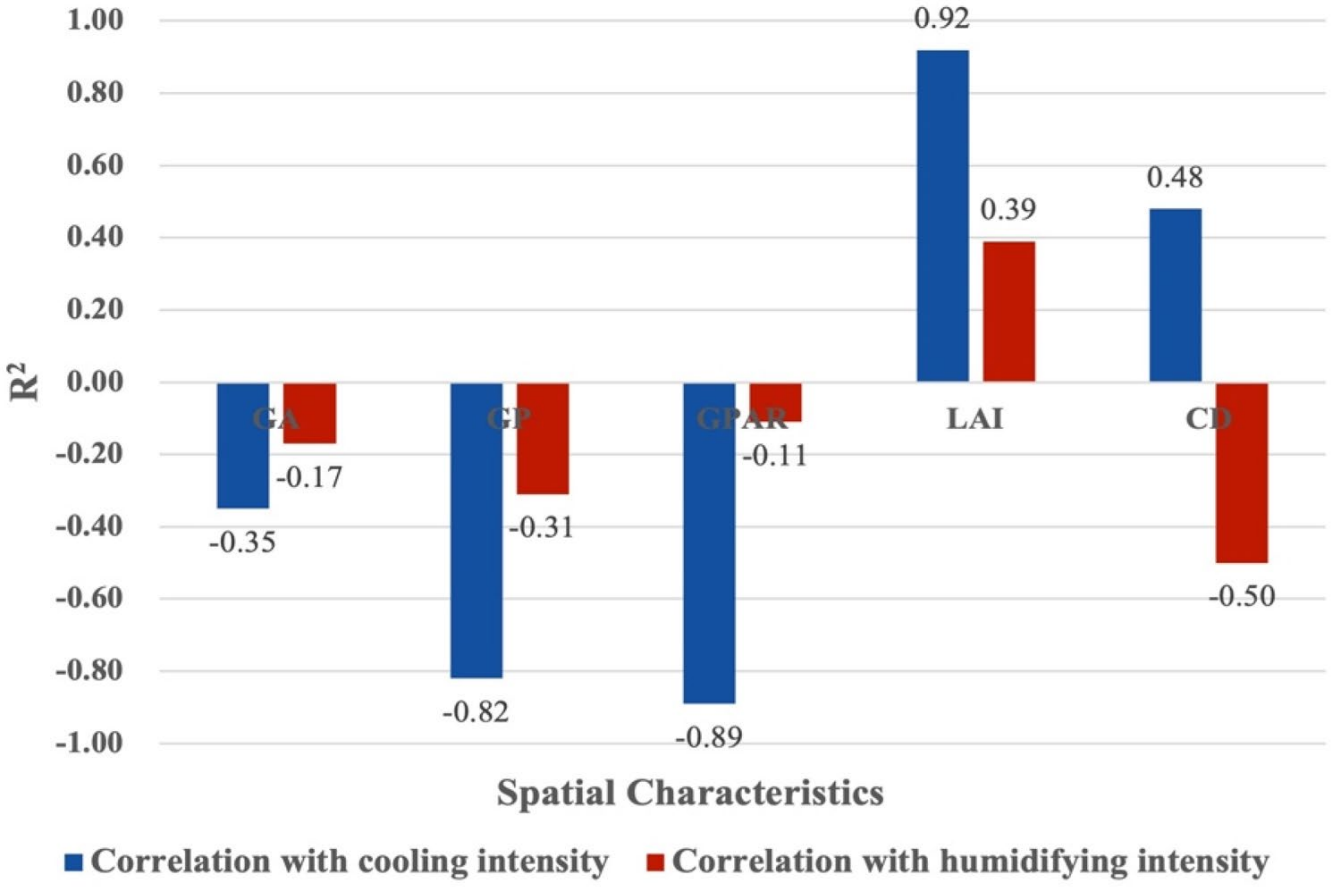
The correlation between the spatial characteristics of large-size green spaces and the intensity of cooling and humidifying (GA means green area; GP means green perimeter; GPAR means green perimeter-area ratio; LAI means leaf area index; CD means canopy density).

Figures 10 and 12 shows the linear regressions between spatial characteristics and cooling intensity in large-size green spaces. There was an insignificant correlation between area and cooling intensity, a weak correlation between canopy density and cooling intensity, and a significant correlation between perimeter, perimeter-area ratio and the leaf area index and cooling intensity. Medium-size green space has the weakest negative correlation between green area and cooling intensity (R^2^ = 0.35), and its leaf area index and cooling intensity have the strongest positive correlation (R^2^ = 0.92). Meanwhile, medium-size green space has weakest negative correlation between perimeter-area ratio and humidifying intensity (R^2^ = 0.11), and its leaf area index and humidifying intensity have most significant positive correlation (R^2^ = 0.39). Figures 10a and 11a show that for every 1 ha increase in area of large-size green spaces, the cooling intensity decreased by 1.02 °C, and the humidifying intensity decreased by 1.22%. Figures 10b and 11b show that for every 100 m increase in perimeter, the cooling intensity decreases by 1.05 °C, and the humidifying intensity decreased by 1.34%. Figures 10c and 11c show that for every 0.005 increase in the perimeter-area ratio, the cooling intensity decreases by 1.43 °C, and each 0.01 increase in perimeter-area ratio, the humidifying intensity decreased by 1.27%. Figures 10d and 11d show that for every 0.1 increase in the leaf area index, the cooling intensity increases by 2.41 °C, and the humidifying intensity increased by 1.37%. Figures 10e and 11e show that each 0.1 increase in the canopy density, the cooling intensity increased by 3.69 °C, and the humidifying intensity decreased by 2.84% (Fig. 12).

### Quantitative analysis of the microclimatic effects of different types of green spaces

#### Quantitative analysis of the effects of different types of green space on cooling intensity

**Figure 13:**
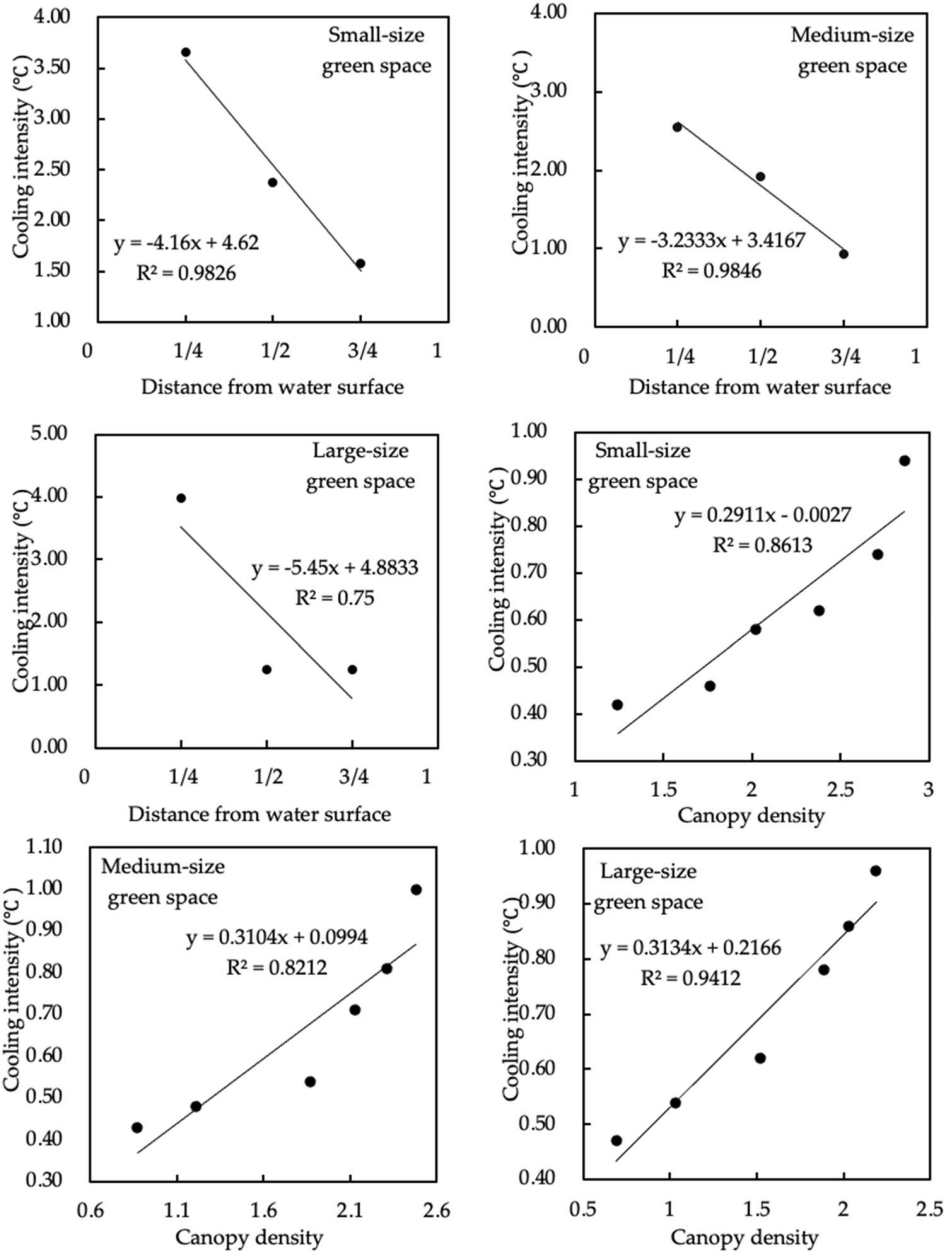
Linear regressions between the distance from different types of green spaces to water areas, canopy density and cooling intensity.

Figure 13 shows the linear regressions between the different types of green spaces and cooling intensity. There were negative correlations between green spaces a short, medium, and long distance from a water body and cooling intensity in small-size green spaces, medium-size green spaces and large-size green spaces. The negative correlation between the distance to a water body and cooling intensity in medium-size green spaces was most significant (R^2^ = 0.985). The greater the distance to a water body, the lower the cooling intensity. For medium-size green spaces, for every 1/4 increase in the distance ratio, the cooling intensity decreased by 0.81 °C. For small-size green spaces, for every 1/4 increase in the distance ratio, the cooling intensity decreased by 1.04 °C. For large-size green spaces, for every 1/4 increase in the distance ratio, the cooling intensity decreased by 1.36 °C. For small-, medium-, and large-size green spaces, there was a positive correlation between canopy density and cooling intensity. There was a most significant positive correlation between canopy density and cooling intensity in large-size green spaces (R^2^ = 0.941). The greater the canopy density, the greater the cooling intensity. For large green spaces, for every 0.5 increase in canopy density, the cooling intensity increased by 0.16 °C. For small-size green spaces, for every 0.5 increase in canopy density, the cooling effect increased by 0.15 °C. For medium-size green spaces, for every 0.5 increase in canopy density, the cooling intensity increased by 0.16 °C.

#### Quantitative analysis of the effects of different types of green space on humidifying intensity

**Figure 14:**
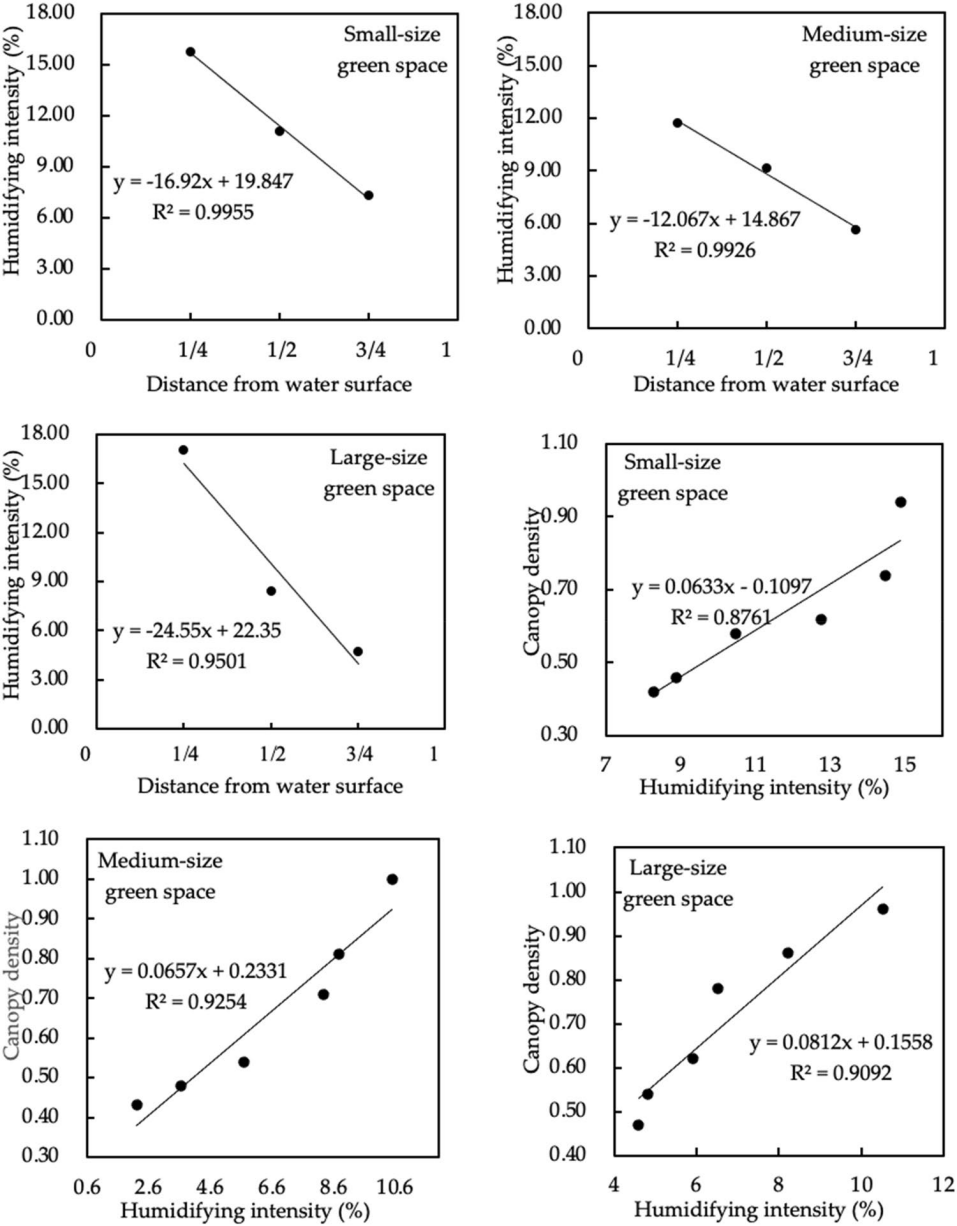
Linear regressions between the distance from different types of green space to water area, canopy density and humidifying intensity.

**Figure 15:**
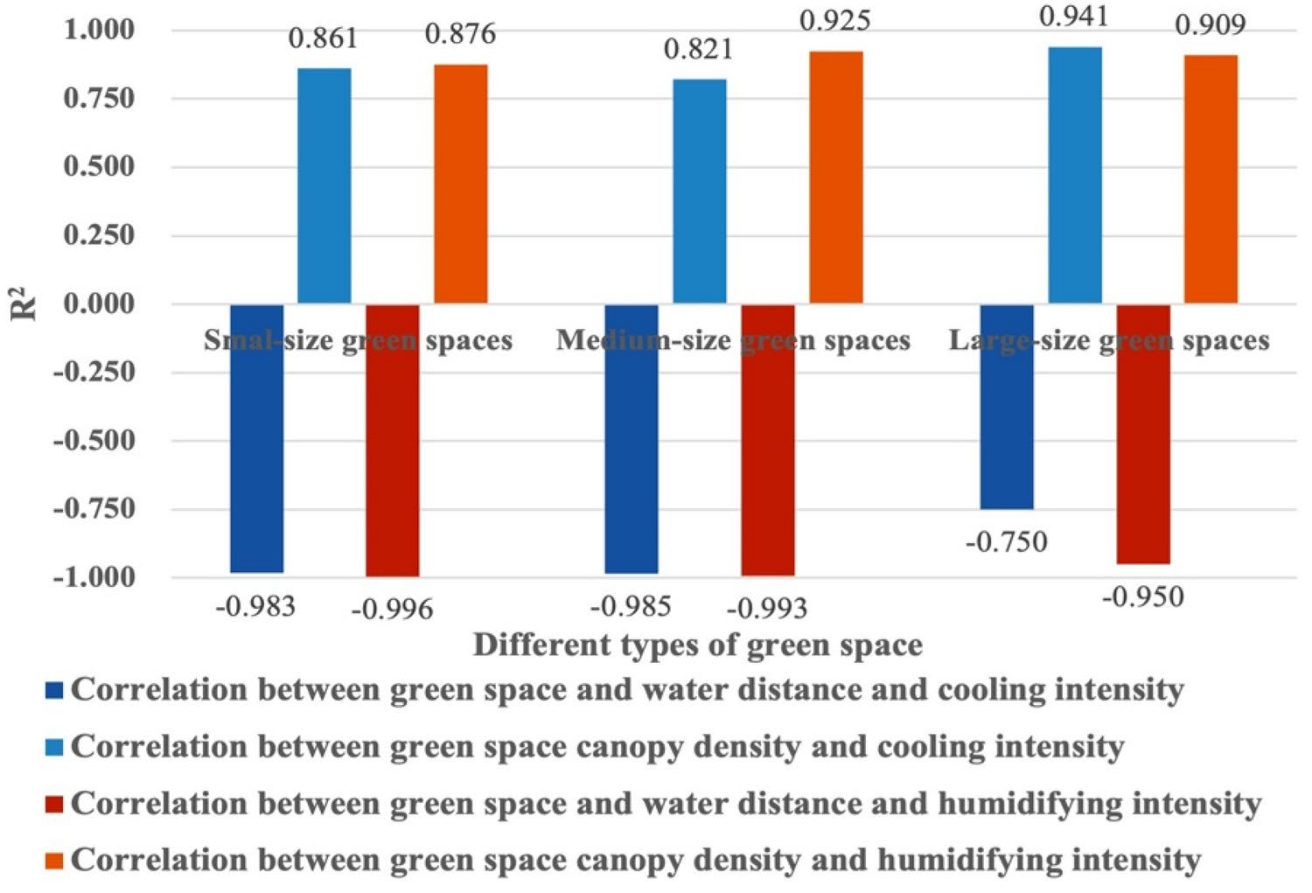
Correlation of different green space types with water distance, canopy density and cooling and humidifying intensity.

Figure 14 shows the linear regression between the distance of a green space from a water body, canopy density and humidifying intensity. There was a negative correlation between the distance to a water body and humidifying intensity in small, medium, and large green spaces. The negative correlation between the distance to a water body and humidifying intensity in small green spaces was most significant (R^2^ = 0.996). The longer the distance, the lower the humidifying intensity. For small green spaces, for every 1/4 in-crease in the distance ratio, the humidifying intensity decreased by 4.23%. For medium-size green spaces, for every 1/4 increase in the distance ratio, the humidifying intensity decreased by 3.02%. For large-size green spaces, for every 1/4 increase in the distance ratio, the humidifying intensity de-creased by 6.14%. For small, medium, and large green spaces, there was a positive correlation between canopy density and humidifying intensity. The positive correlation between canopy density and humidifying intensity in medium-size green spaces was extremely significant (R^2^ = 0.925). The greater the canopy density, the greater the humidifying intensity. For medium-size green spaces, for every 0.5 increase in canopy density, the humidifying intensity increased by 3.29%. For small-size green spaces, for every 0.5 increase in canopy density, the humidifying intensity increased by 3.17%. For large-size green spaces, for every 0.5 increase in canopy density, the humidifying intensity increased by 4.06% (Fig. 15).

### Effect of shape and area of water bodies on microclimatic effects based on numerical simulation

#### Banded water

**Figure 16:**
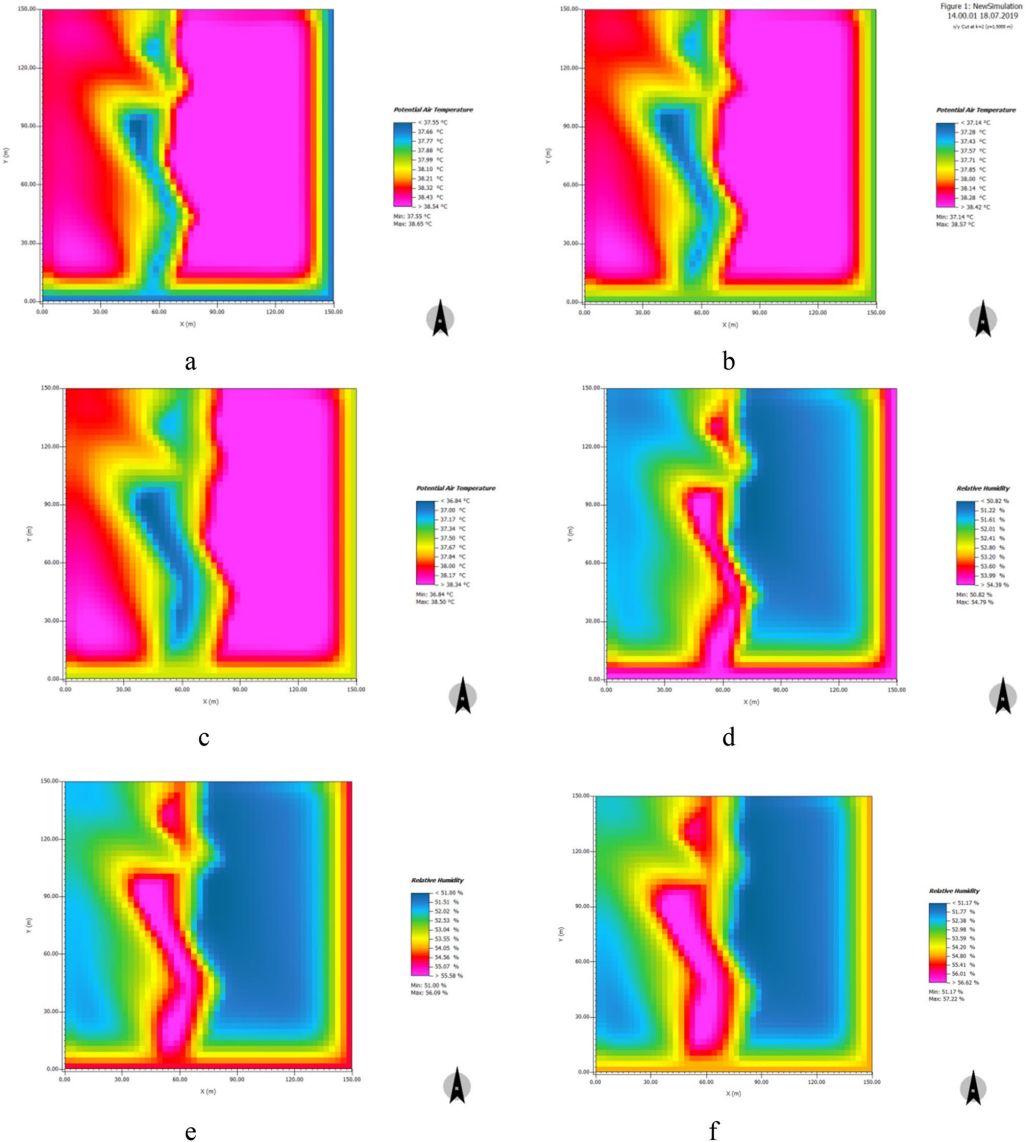
Distribution characteristics of cooling and humidifying effects of simulated increase of banded water area at 14:00. (**a**) original cooling effect of banded water in the sample area; (**b**) cooling effect of 5% increase in water area; (**c**) cooling effect of 10% increase in water area; (**d**) original humidifying effect of banded water in the sample area; (**e**) humidifying effect of 5% increase in water area; (**f**) humidifying effect of 10% increase of water area.

We constructed a numerical simulation model to explore the effects of a simulated increase in water body area on cooling and humidification. Figure 16 shows the simulated distribution characteristics of temperature and relative humidity after a 5% and 10% increase in water area at 14:00 when temperatures were high. The results suggest that between 7:00 and 10:00, with a 5% and 10% increase in water area, the air temperature was basically the same and the cooling effect was insignificant. However, between 12:00 and 19:00 and particularly in the hours between 13:00 and 16:00 when temperatures were highest, a 5% increase in water area produced a significant cooling effect, with a daily average value of 0.05 °C and a maximum value of 0.09 °C. A 10% increase in water area produced an extremely significant cooling effect, with a daily average value of 0.07 °C and a maximum value of 0.14 °C. From 11:00 to 19:00, a 5% increase in water area produced a significant humidifying effect, with a daily average value of 0.08% and a maximum value of 0.17%. A 10% increase produced an extremely significant humidifying effect, with a daily average value of 0.13% and a maximum value of 0.26% (See supplementary file).

#### Massive water

**Figure 17:**
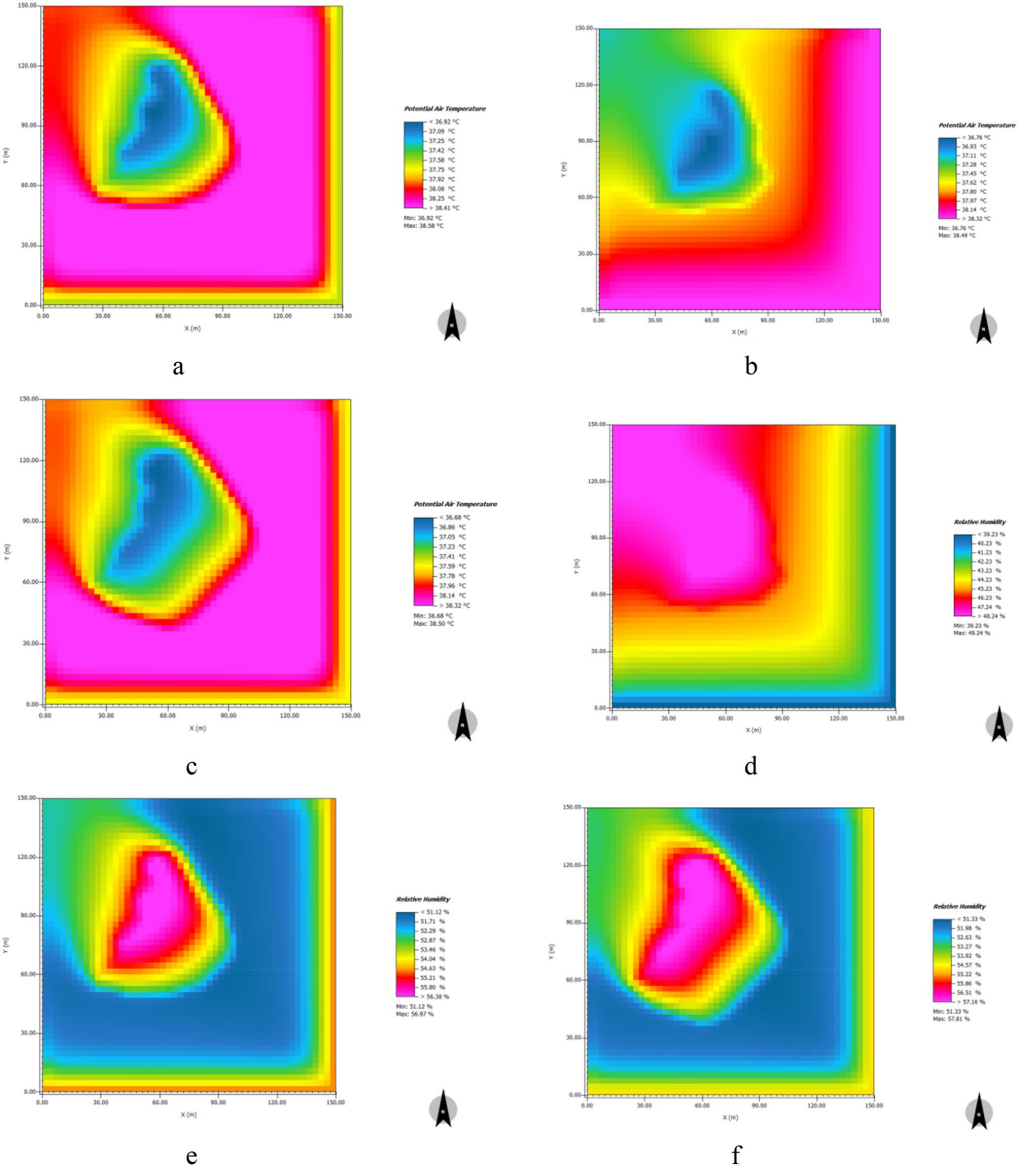
Distribution characteristics of cooling and humidifying effects of simulated increase of massive water area at 14:00. (**a**) original cooling effect of massive water in the sample area; (**b**) cooling effect of 5% increase in water area; (**c**) cooling effect of 10% increase in water area; (**d**) original humidifying effect of massive water in the sample area; (**e**) humidifying effect of 5% increase in water area; (**f**) humidifying effect of 10% increase of water area.

Figure 17 shows the simulated distribution characteristics of the cooling and humidifying effects after a 5% and 10% increase in the water area at 14:00 when temperatures were high. Between 8:00 and 19:00, a 5% and 10% increase in water area produced a significant cooling effect. At 19:00, the numerical simulation result was abnormal when the water area increased by 5% and 10%; at 13:00, the numerical simulation result was also ab-normal when the water area increased by 10%. After excluding the abnormal simulated data, a 5% increase in water area produced a cooling effect, with a daily average value of 0.06 °C and a maximum value of 0.10 °C. A 10% increase in water area produced an extremely significant cooling effect, with a daily average value of 0.10 °C and a maximum value of 0.18 °C. Between 11:00 and 19:00, a 5% increase in water area produced a significant humidifying effect, with a daily average value of 0.05% and a maximum value of 0.13%. A 10% increase in water area produced an extremely significant humidifying effect, with a daily average value of 0.13% and a maximum value of 0.27% (See supplementary file).

#### Annular water

**Figure 18:**
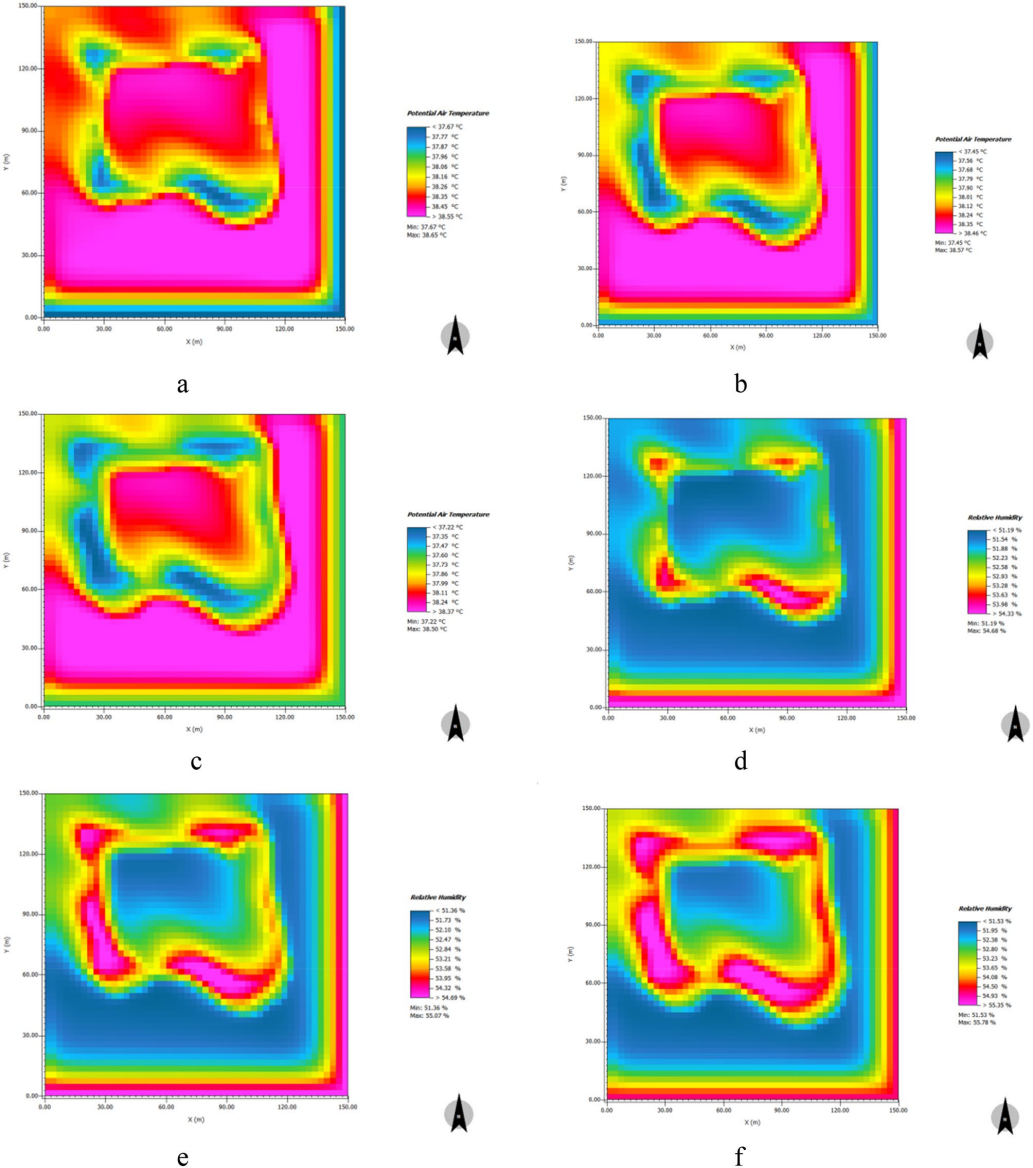
Distribution characteristics of cooling and humidifying effects of simulated increase of annular water area at 14:00. (**a**) original cooling effect of annular water in the sample area; (**b**) cooling effect of 5% increase in water area; (**c**) cooling effect of 10% increase in water area; (**d**) original humidifying effect of annular water in the sample area; (**e**) humidifying effect of 5% increase in water area; (**f**) humidifying effect of 10% increase of water area.

Figure 18 shows the simulated distribution characteristics of the cooling and humidifying effects after a 5% and 10% increase in the area of the annular water body at 14:00 when temperatures were high. Between 7:00 and 19:00, a 5% and 10% increase in water area produced a significant cooling effect. Between 11:00 and 16:00 when temperatures were high, a 5% increase in water area produced a cooling effect, with a daily average value of 0.06 °C and a maximum value of 0.14 °C°C and a 10% increase in water area produced an extremely significant cooling effect, with a daily average value of 0.13 °C and a maximum value of 0.28 °C. Between 7:00 and 19:00, a 5% and 10% increase in water area produced significant humidifying effects. Between 11:00 and 16:00 when temperatures were high, a 5% increase in water area produced an extremely significant humidifying effect, with a daily average value of 0.17% and a maximum value of 0.39% and a 10% increase in water area produced an extremely significant humidifying effect with a daily average value of 0.38% and a maximum value of 0.81% (See supplementary file).

## Discussion

In this study, we analyzed the correlations between the size/type of green spaces and microclimatic effect, the influence of woodland density on the microclimatic effect, the effects of water bodies on the microclimatic effect, and the correlation between the shape and area of water bodies and the microclimatic effect. We observed that the cooling and humidifying effects of green spaces in urban environments do not necessarily increase with an increase in green space area. Although the average daily temperature variations in small-size green spaces were large, the cooling and humidifying effects of small-size green spaces were more significant than those of large-size green spaces. This result is similar to the results reported by Chen et al. ^7^, Su et al. ^5^ and Chang et al. ^6^. We found that the cooling and humidifying effects of medium-size green spaces were greater compared with small-size green spaces and large-size green spaces. According to Zhu et al. ^23^, when the canopy density of an urban green space reaches 10%, the green space produces a certain cooling and humidifying effect; the greater the canopy density, the more significant its cooling and humidifying effects. When the canopy density reaches 70%, the cooling and humidifying effect of green spaces tend to stabilize ^3^. A previous study suggested that the area of green space greatly affects the cooling and humidifying effects, but beyond a certain threshold, these effects were not enhanced with an increase in green space area. Therefore, we chose to research the influence of structural characteristics (i.e., perimeter, area, perimeter-area ratio, canopy density and average leaf area index, etc.) of different types of green spaces on their cooling effect by dividing the green spaces into small-, medium-, and large-size green spaces.

Previous research suggested that air temperature increases gradually, and humidity decreases gradually with an increase in distance from a water body. Furthermore, water bodies within a park also produce a certain cooling and humidifying effect on the local environment ^24–26^. We reached similar conclusions in this study. However, there have been relatively few studies focusing on the relationship between the distance from a water body and the cooling and humidifying effects and the quantitative relationship between the shape and area of water bodies and the cooling and humidifying effects in green spaces. Therefore, we further divided green spaces into several types: green spaces a short, medium, and long distance from water. For medium-size green spaces, for every 1/4 increase in the distance ratio, the cooling intensity decreased by 0.81 °C and the humidifying intensity decreased by 3.02%. For small-size green spaces, for every 1/4 increase in the distance ratio, the cooling intensity decreased by 1.04 °C and the humidifying intensity decreased by 4.23%. For large-size green spaces, for every 1/4 increase in the distance ratio, the cooling intensity decreased by 1.36 °C and the humidifying intensity decreased by 6.14%. The shape of the water body (banded water area, massive water area and annular water area) also had an effect: for every 5% increase in banded water area, the cooling effect increased by about 0.02 °C and the humidifying effect by about 0.05%; for 5% every increase in massive water area, the cooling effect increased by about 0.04 °C and the humidifying effect by about 0.08%; for every 5% increase in annular water area, the cooling effect increased by about 0.07 °C and the humidifying effect by about 0.21%. The magnitude of the cooling and humidifying effects ranked in the following order: annular water area > massive water area > banded water area. The result further revealed that green spaces with a water body have enhanced cooling and humidifying effects due to the large thermal capacity of the water area, lower average reflectivity of solar radiation and greater transmissivity of solar short-wave radiation ^17,27^. These factors are the main reasons for the significant correlation between the distance to a water body and the cooling and humidifying effects and the significant influence of the shape and size of water bodies on local cooling and humidification. Further research is required to analyze these relationships in more detail.

However, there have been relatively few studies on the influence of woodland density in parks on the cooling and humidifying effects. Result shows that for every 0.5 increase in canopy density in large-size green spaces, the cooling intensity increased by 0.16 °C; for every 0.5 increase by in canopy density in small-size green spaces, the cooling intensity increased by 0.15 °C; for every 0.5 increase in canopy density in medium-size green spaces, the cooling intensity increased by 0.16 °C. Our results showed a significant influence of canopy density on cooling and humidification. Further research should be carried out to quantify in more detail the relationship between woodland density and the cooling and humidifying effects of green spaces.

Since the perimeter-area ratio controls the shape of a park, the smaller the ratio and the more round-like the shape, the more stable the internal space of a park. Studies have shown that the shape of the patch can significantly affect the stability of the interior of the green space. The closer the aspect ratio of the patch to 1:1 or the more circular, the more stable the interior space of the plant. If the internal space of green plants has significant stability, the branches and leaves in the plant community will form a shady canopy. It not only blocks and reflects direct solar radiation, but also blocks reflected heat from buildings and other adjacent objects, thereby reducing long-wave radiation heat from the ground. Therefore, the light intensity transmitted into the community will inevitably affect the temperature and humidity effect of plants to a certain extent, which has a positive effect on blocking the entry of external solar radiation and regulating the temperature of its own internal space, and finally makes the green space have a cooling and humidifying effect. Thus, it effectively regulates the internal air temperature of the park. Accordingly, the cooling and humidifying effects are stronger. With a greater park area, leaf area index, canopy density, and plant density, the cooling and humidifying effects become stronger. The four variables (green area, perimeter-area ratio, leaf area index, and canopy density) explain 61.9% of the cooling effect and 61.2% of the humidifying effect on average. However, other factors which also in-fluence the cooling and humidifying effect of green spaces include the geographical lo-cation of urban green spaces, type of land use, type of impervious pavement, the spatial layout of arbors, surrounding urban morphology, and external environment. Further research on these aspects should be conducted in the future.

The conclusions of this study are verified as: by using SPSS software to analyze the correlation between the spatial structure characteristics of different green space types and their cooling and humidification effects, a prediction model is constructed. To further verify the reliability of the above models, the "cross-validation method" was used to verify the multiple regression models of the cooling range and humidification range respectively, and the verification results proved the reliability of the prediction model. In this study, the air temperature and relative humidity were numerically simulated and analyzed according to the root mean square error RMSE of 0.14 °C and 0.95%, and the mean absolute percentage error of MAPE of 1.56% and 2.69%, respectively. The simulation analysis results show that the research conclusions are valid and reliable.

The results of this study are only applicable to similar areas with Suzhou in terms of vegetation type, climate type and relationship with water bodies. Any generalizations beyond this that have not been shown to be sufficiently reliable to support landscape planning and design decisions are as follows:

1. The cooling and humidifying effect of green space on the local urban environment does not increase with the increase of the area. Although the daily average temperature of small green spaces varies greatly, the effect of cooling and humidification is more significant than that of large green spaces.
2. Compared with small green space and large green space, the cooling and humidifying ability of medium green space is more significant.
3. The distance between medium-sized green space, small green space and large green space and the water area increases proportionally, while cooling and humidification decrease.
4. The water area of different shapes in the park increase proportionally, and the cooling and humidification will increase.

The findings of this study may be weakly relevant that they cannot be trusted in decision making may be the correlation between the spatial characteristics of medium-sized green space, small green space, and large-scale green space and the range of cooling and humidification is weak and may not be trusted in decision-making. Moreover, we believe that the total sample size of the 29 park green spaces in SIP is limited, so this study has some limitations that can be improved in future studies.

## Conclusions

Since the sampled sites were mainly located in a subtropical maritime monsoon climate zone and the tests were mainly conducted in the summer, the results apply only to this season and climatic zone. Based on daily trends in the cooling and humidifying effects of green spaces of different sizes and the influence of self-structure factors of green spaces (i.e., area, perimeter, perimeter-area ratio, leaf area index, and canopy density) on cooling and humidification, we further researched the influence of woodland density and water bodies, to provide an accurate reference for the systematic planning of urban green spaces. The results are summarized below:

1. Between 10:00 and 16:00, the average cooling effect of the different types of green spaces ranked in the following order: medium-size green space > large-size green space > small-size green space; the average humidifying effect ranked as follows: medium-size green space > small-size green space > large-size green space. The cooling and humidifying effects were greatest in medium-size green spaces. Furthermore, the cooling and humidifying effects of the different types of green spaces varied at different hours, and their cooling and humidifying effects on local urban environments did not significantly increase with an increase in green space area.
2. There was a significant positive correlation between canopy density and cooling intensity in small-size green spaces. The leaf area index, perimeter-area ratio, area and canopy density explained about 59.3% of the cooling effect and 61.2% of the humidifying effect of small-size green spaces. For medium-size green spaces, there were significant positive correlations between green space area, perimeter-area ratio and cooling intensity, and the positive correlation between area and the humidifying intensity was greatest. Green space area, perimeter-area ratio, leaf area index and canopy density explained about 70.7% of the cooling effect and about 71.1% of the humidifying effect of medium-size green spaces. For large-size green spaces, the correlations between the leaf area index, perimeter-area ratio and the cooling in-tensity were greatest and the correlation between canopy density and humidifying intensity was greatest. Canopy density, leaf area index and perimeter-area ratio of green spaces explained about 55.8% of the cooling effect and about 51.3% of the humidifying effect of large-size green spaces.
3. The magnitude of the effect of woodland density on the cooling and humidifying effects ranked as follows: spaces with high woodland density > spaces with low woodland density.

Previous studies concluded that larger parks induce better cooling and humidifying effects. However, in this study, medium-sized green spaces produced greater cooling and humidifying effects than small-size and large-size green spaces. We showed that the cooling effect of a park would improve further with a greater park area, a more rectangular or round-like shape, a greater density of plants, annular-shaped design of water areas within the green space, greater area of water body, more complex boundary of green patches, more simplified external boundary of parks, and scattered arrangement of woodland and hard texture, which is of great practical significance for guidance on systematic planning of urban green spaces.

## Supplementary Information

Supplementary Figures.

## Data Availability

All data generated or analysed during this study are included in this published article.
