# Supplementary material for: Influence of spatial characteristics of green spaces on microclimate in Suzhou Industrial Park of China
Source: Sci Rep. 2022 Jun 1;12:9121. doi: 10.1038/s41598-022-13108-1 (PMC9160017; doi:10.1038/s41598-022-13108-1)
Supplement: Supplementary file 1 — Supplementary Figures. [file 41598_2022_13108_MOESM1_ESM.pdf]

Appendices

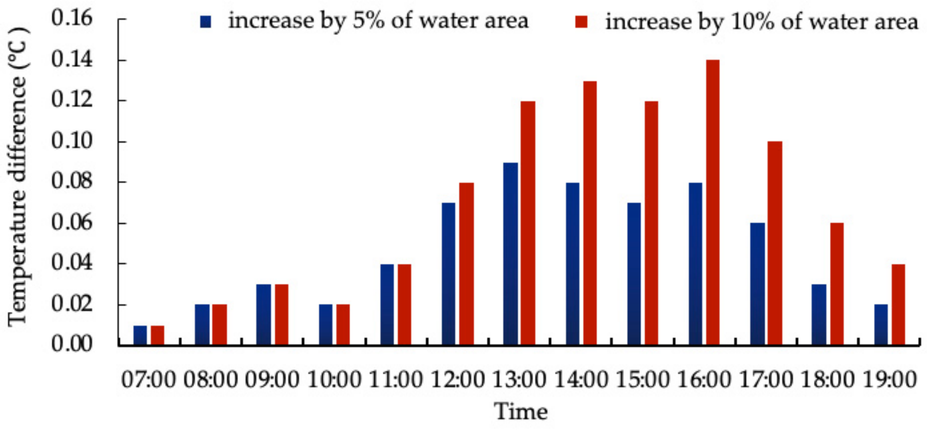

Figure 1 Differences in temperature in different simulated areas of banded water

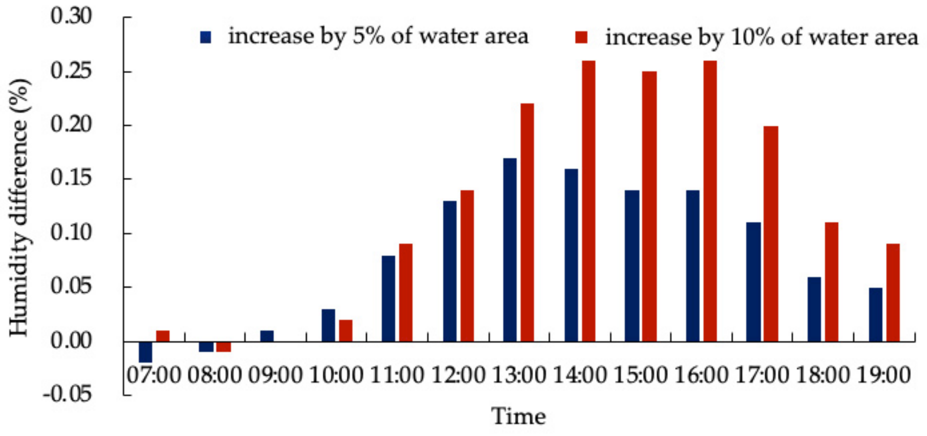

Figure 2 Differences in humidity in different simulated areas of banded water

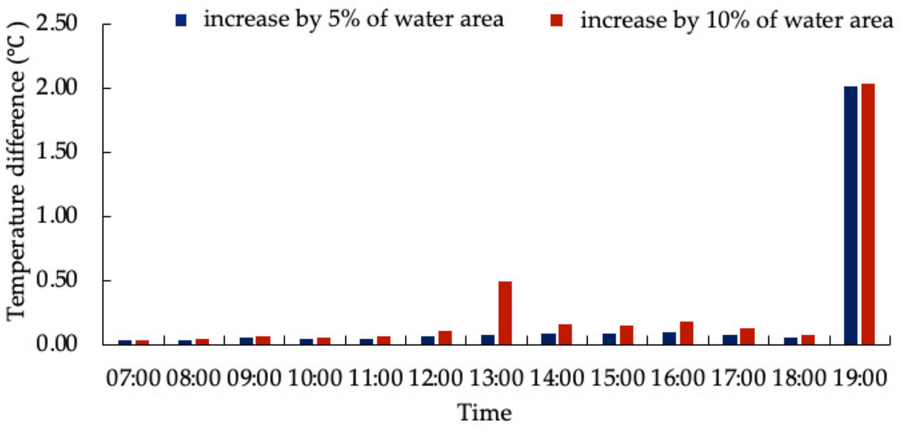

Figure 3 Differences in temperature in different simulated areas of massive water

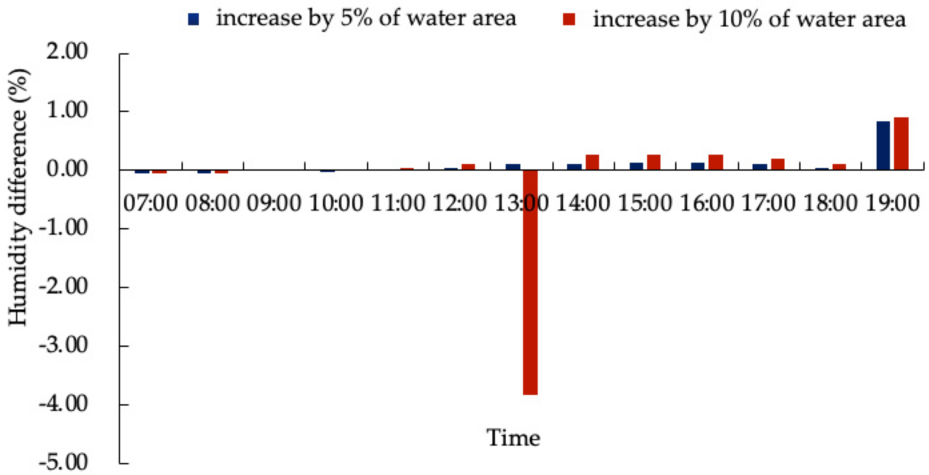

Figure 4 Differences in humidity in different simulated areas of massive water

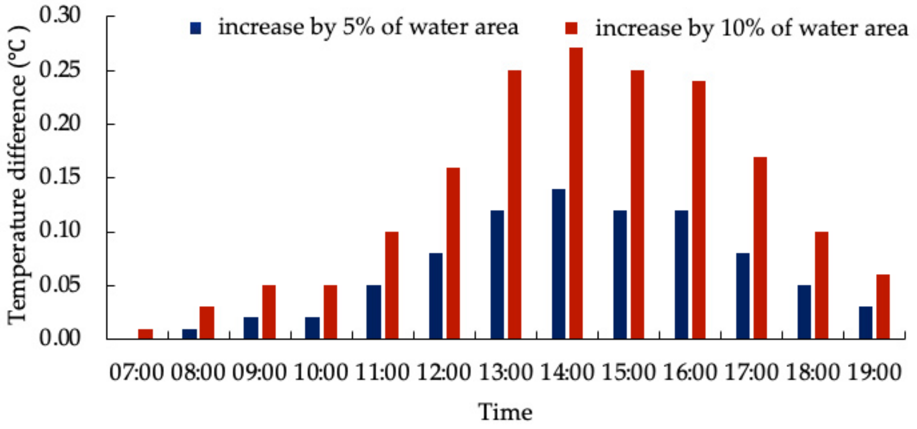

Figure 5 Differences in temperature in different simulated areas of annular water

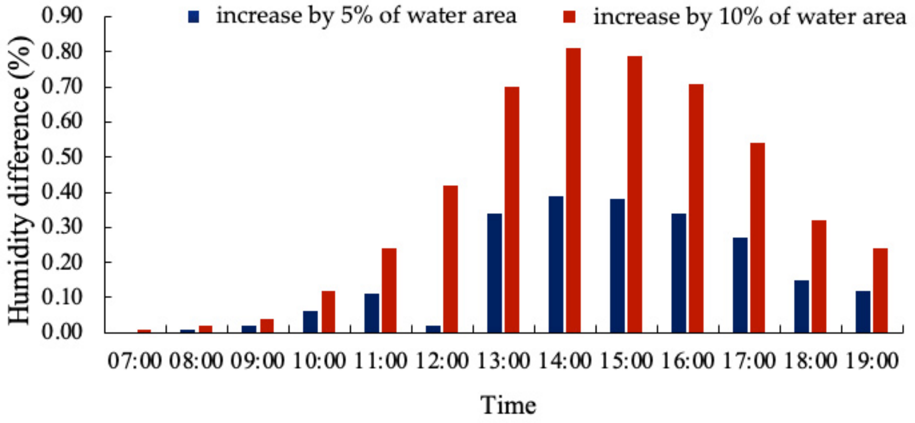

Figure 6 Differences in humidity in different simulated areas of annular water
